# Integrative machine learning-guided *in silico* and *in vitro* approach reveals selective small molecule inhibitors targeting mutant IDH1

**DOI:** 10.1039/d5ra06290j

**Published:** 2025-12-18

**Authors:** Mayank Bajaj, Rohit Kumar, Vishal Pandey, Sahanawaz Parvez, Hemanth Kumar Tanneru, Polamarasetty Aparoy, Roy Karnati

**Affiliations:** a Translational Biology Laboratory, Department of Animal Biology, School of Life Sciences, University of Hyderabad Hyderabad – 500046 Telangana India roykarnati@uohyd.ac.in +91-9652921092; b Molecular Modelling and Protein Engineering Lab, Indian Institute of Petroleum and Energy Visakhapatnam – 530003 Andhra Pradesh India aparoy.bio@iipe.ac.in +91-8219241123; c Department of Chemical Engineering, Indian Institute of Petroleum and Energy Visakhapatnam – 530003 Andhra Pradesh India

## Abstract

Mutations in Isocitrate Dehydrogenase 1 (IDH1) are found in over 80% of WHO grade II/III gliomas. These mutations, specifically R132H, confer a neomorphic activity that converts α-ketoglutarate (α-KG) into the oncometabolite 2-hydroxyglutarate (2HG), a key driver in glioma pathogenesis. While the therapeutic potential of targeting mutant IDH1 (MT-IDH1) is established, the discovery of novel and selective inhibitors remains a priority. Leveraging the growing availability of pharmacological data, we developed regression-based ML models to predict pIC_50_ values and identify potent inhibitors of MT-IDH1. We trained these models using a dataset of 1631 compounds from ChEMBL, utilising 208 molecular descriptors derived from RDKit. Among the three algorithms evaluated, the Random Forest model demonstrated superior performance, achieving high predictive accuracy on the training set and robust generalisability on the test set. Feature importance analysis provided critical insights related to lipophilicity, halogen, and electronic factors as the key molecular determinants of inhibitor activity. We subsequently deployed this model to screen drug databases, identifying five promising hits. These candidates were further validated through *in silico* molecular docking, molecular dynamics simulation, and MM/PBSA free energy calculations. Experimental *in vitro* enzymatic assays confirmed that these compounds selectively inhibit MT-IDH1 with IC_50_ values in the micromolar range, while exhibiting no significant activity against the WT-IDH1. While the mechanism of action of these compounds as IDH inhibitors is yet to be established, our results support these compounds as potent and selective hits. They offer a promising foundation for structural optimisation and the development of next-generation therapeutics against MT-IDH1 malignancies.

## Abstract 2

The study developed machine learning models to predict the key hotspots within the allosteric inhibitor binding site of mutant Isocitrate Dehydrogenase 1 (IDH1) enzyme and validated the inhibition by *in silico* and *in vitro* screening.

## Graphical abstract

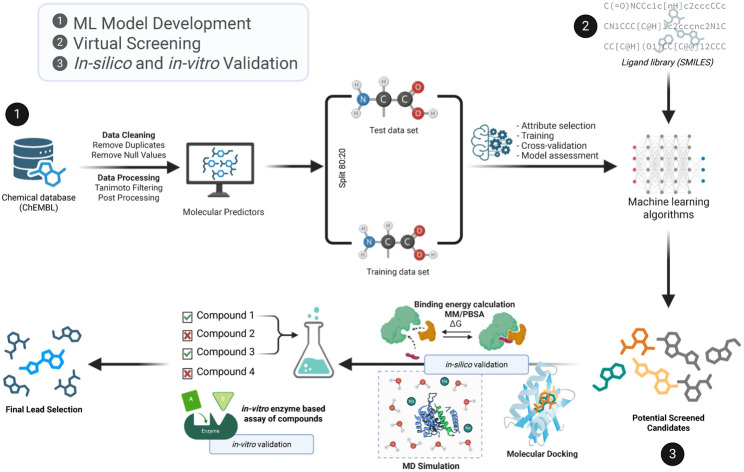

## 1 Introduction

In humans, the isocitrate dehydrogenase (IDH) enzyme is encoded by five different IDH genes (IDH1, IDH2, IDH3A, IDH3B, and IDH3C). IDH1, situated on the 2q33.3 gene locus, is involved in the decarboxylation of isocitrate (ICT) into 2-ketoglutarate, also known as α-ketoglutarate (α-KG). This process generates NADPH from NADP^+^. Reductive carboxylation converts α-KG back to ICT, where NADPH is consumed. The IDH2 gene on 15q26.1 performs the same reversible process as IDH1 within the mitochondria.^1^ IDH1 and IDH2 work as homodimers with similar amino acid sequences and structural properties.^2–5^ IDH1 and IDH2 both play an essential role in forward oxidative decarboxylation, an important cellular defence mechanism against oxidative damage and act as a source of NADPH and aid in reductive synthesis.^6,7^ Furthermore, these isoforms influence the function of dioxygenase enzymes by generating α-KG, a critical substrate for these enzymes.^8^ IDH1 and IDH2 reverse reductive carboxylation reactions are also essential for many biological activities. It regulates lipogenesis and glycolysis by promoting ICT production. This complex interaction between IDH1, IDH2, and the reverse reductive carboxylation pathway substantially contributes to cellular metabolic control and function.^9–11^ IDH3, the third member of the IDH enzyme family, is an essential enzyme in the tricarboxylic acid (TCA) cycle. IDH3, unlike the previously described IDH1 and IDH2, catalyses the irreversible conversion of ICT into α-KG, generating NADH. The NADH produced contributes to the generation of ATP *via* the electron transport chain.^12^ IDH3 regulation is determined mainly by substrate availability and the presence of positive allosteric effectors, such as calcium, citrate, and ADP, and negative effectors like NADH, NADPH, and ATP.^13^

Significant studies have been made on the knowledge of frequent mutations in the IDH1 and IDH2 genes at somatic level, highlighting their relevance in oncogenesis. IDH1 and IDH2 mutations are prevalent in low-grade gliomas (LGG), accounting for more than 70% of cases. 5% to 14% of primary glioblastomas, a subtype of glioblastoma, and secondary glioblastomas generated from diffuse astrocytoma (WHO CNS grade 4) have also been shown to carry these mutations, with rates ranging from 55% to 88%. In cartilaginous and bone tumours, the prevalence of these mutations ranges from 20% to 80%, while 6% to 30% in intrahepatic cholangiocarcinoma, a malignant liver tumour, and 15% to 30% in acute myeloid leukaemia (AML). Although less common, these mutations have also been reported in myelodysplastic syndrome, angioimmunoblastic T-cell lymphoma, and other solid tumours.^14–16^ The existence of these mutations inside the enzymes' critical catalytic domain is interesting since they result in unusual catalytic properties that generate oncometabolites and activate carcinogenic pathways. The precise impact of these mutations on the enzyme function and downstream signalling pathways is a primary focus of cancer research.^17,18^

The mutations observed in IDH1 and IDH2 are heterozygous somatic missense substitutions known to play a central role in gaining new functions over the wild-type enzyme. These recurrent mutations exclusively affect specific arginine residues within the active sites, substituting different amino acids. These arginine residues are located at precise hotspots, including R100Q, R132H, R132L, R132S, R132G, and R132C in IDH1, as well as R140Q and R172K in IDH2. Unlike IDH1 and IDH2, no tumour-related mutations were detected in IDH3 genes.^19^ The R132H mutation is the most common in IDH1, accounting for roughly 89% of documented mutations. The other IDH1 mutations occur at lower rates, emphasising their limited contribution to the overall mutational landscape.^20^

The arginine amino acid sequence at R132 within IDH1 is vital for the active site's catalytic function. It is situated within the ICT binding pocket, where it orchestrates the transformation of NADP^+^ into NADPH, resulting in α-KG production. The alteration of R132H imparts distinct characteristics to the IDH1 enzymatic activity. This dysregulated enzymatic function disrupts the normal cellular metabolic balance, contributing to oncogenesis and cancer progression.^20,21^ These mutations result in acquiring a catalytic neomorphic activity, converting α-KG to a unique endogenous oncometabolite called 2-hydroxyglutarate (2HG), predominantly yielding the optically active D-form of 2HG. These mutations are responsible for the abnormal production of 2DHG, which further contributes to epigenetic dysregulation, influencing oncogenesis and cancer development.^22–24^

IDH inhibitors are being investigated as a potential treatment for these cancers. Ivosidenib (AG-120), an important IDH1 inhibitor, has emerged as a promising candidate capable of targeting various IDH1 mutants. It has successfully progressed to phase III clinical trials in several indications, including cholangiocarcinoma, acute myeloid leukaemia (AML), and other solid tumours.^25^ Other examples of MT-IDH1 inhibitors include olutasidenib (FT-2102), IDH305, vorasidenib (AG-881), GSK2857916, and BAY1436032. These inhibitors are still in the early stages of development and clinical trials. Most highly effective R132H IDH1 inhibitors, whose crystal structures have been studied, exhibit inhibition through an allosteric mechanism rather than the conventional active-site binding mode. Considering the structural variety observed, these allosteric inhibitors bind at the dimer interface, revealing an intriguing aspect of their inhibitory action. The diversity in their structural features suggests a rich landscape of potential binding sites and modes, which can be further explored and harnessed in developing novel and targeted IDH1 inhibitors.^26–28^

Computer-aided drug discovery (CADD) methodologies are gaining growing interest due to their potential to address the challenges associated to scale, time, and cost as in traditional experimental methods.^29,30^ This computational approach to biological sciences has been successful in bringing new drug compounds for various diseases such as COVID-19, HIV and cancer.^31–33^ Numerous CADD methodologies have emerged, incorporating machine learning techniques to enhance the precision and effectiveness of these methods.^34^ Artificial intelligence (AI) and machine learning (ML) have recently been advanced in anticancer drug discovery, enabling precise target identification and compounds through advanced algorithms, offering a paradigm shift in drug discovery process. These systems analyse complex biological data to classify, cluster, and predict network patterns, improving the accuracy and efficiency of biological data analysis.^35^ In drug discovery, ML can be used effectively in drug design, chemical synthesis, drug screening, pharmacology and drug repurposing. Virtual screening, for example, relies on diverse algorithms, including nearest-neighbour classifiers, support vector machine, and deep neural networks to assess the synthetic feasibility and predict *in vitro* activity and toxicity, enhancing the efficiency of the screening process.^36^ Only a few studies have reported methodologies for drug inhibitory activity prediction, suggesting an extensive gap to be filled with an effective computational prediction model for MT-IDH1 specific inhibitor response. Here, our study aims to provide a robust ML model for screening and predicting the IC_50_ value of potential IDH1 inhibitors from chemical structures. The ML model, together with known drug discovery methods can be used for virtual screening of potential candidates against MT-IDH1. Building on this, we screened and identified five compounds using the RF model and studied their potential effect on MT-IDH1 inhibition. We used olutasidenib^28^ and α-mangostin^37^ as positive control drugs which are established MT-IDH1 inhibitors. Temozolomide, an important chemotherapy drug and an alkylating agent known to work in gliomas by generating reactive methylating species that alkylate DNA (mostly O_6_-guanine), leading to tumour inhibition was used as an inactive or non-inhibitor negative control for *in silico* studies.^38,39^ Its mechanism of action is very different from that of MT-IDH1 inhibitors. *In silico* drug discovery methods like molecular docking, molecular dynamics simulations, and free energy calculations were employed for all the molecules. Further, *in vitro* enzyme activity assays on IDH1 wildtype and mutant were used to validate the inhibitory potential of screened compounds. Further studies are required to decipher their mode of inhibition in gliomas, and their strong potential for further structural optimisation and development as effective therapeutic candidates for MT-IDH1 cancers.

## 2 Materials and methods

### 2.1 Data collection and preparation

The initial dataset comprising SMILES information and corresponding half-maximal inhibitory concentration (IC_50_) values was obtained from the ChEMBL database^40^ (last accessed July 13, 2025). The IC_50_ was selected as the primary measure of biological activity, representing the concentration of a compound required to inhibit the target protein's activity by 50%. The initial data collection consisted of 4439 entries. Specific reported IC_50_ values were used for each compound, enabling the calculation of negative logarithmic scale, pIC_50_. This transformation helps linearise the data range, reduces skewness caused by orders-of-magnitude differences in potency, and provides a robust, continuous training label that directly correlates with binding affinity. The choice for IC_50_ metric in model development was so as unlike binary classifications (active/inactive), IC_50_ provides a continuous, quantitative gradient of bioactivity. For modeling purposes, these values were normalized to pIC_50_, allowing the machine learning algorithms to learn subtle structure–activity relationships across the potency landscape. The dataset was then cleaned (BAO filter, assay single protein) and prepared by removing the compounds that were duplicated (based on structures of SMILES) or lacked activity (null based on nM standard units) information. A total of 1631 molecules (SI File S1) were used for model building and validation. To ensure molecular diversity and minimise overfitting, the Tanimoto coefficient, also known as the Jaccard index was employed to filter out highly similar compounds based on their binary molecular fingerprints. It is a widely used metric in cheminformatics that quantifies pairwise molecular similarity on a 0 to 1 scale, with values ≥0.8 typically indicating high structural resemblance. It is particularly well-suited for fingerprint-based similarity calculations in virtual screening, quality structure activity relationship (QSAR) modelling, and diversity analysis.^41^ To mitigate structural redundancy, Morgan fingerprints (radius 2, 2048 bits) were generated and employed to construct a similarity graph based on the Tanimoto coefficient. Molecules exhibiting a Tanimoto similarity ≥0.85 were defined as connected components. This threshold was selected to strike a balance between maintaining chemical diversity and preserving dataset size. For each connected component (cluster of highly similar compounds), the molecule with the highest pIC_50_ was retained. The representative chemotype with the lowest ChEMBL ID serves as a tie-breaker, while other molecules were discarded. This keeps one representative from each near-duplicate group while favouring the most active chemotype.

**Fig. 1:**
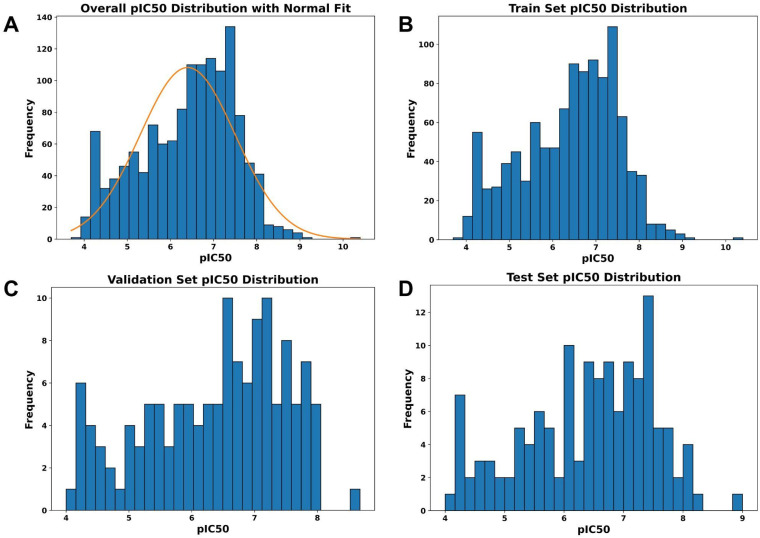
Distribution of pIC_50_ for the (A) full dataset and for each individual data split, (B) training, (C) validation, and (D) test. The comparable distribution shapes across splits demonstrate that the stratified sampling procedure preserved the underlying activity distribution, enabling fair evaluation and enhancing model generalizability.

This protocol reduced the dataset for the final modelling from 1631 compounds to 1342 molecules, providing enough data while also avoiding redundancy. (SI File S2). To make sure that every subset of our data represents the full range of pIC_50_ values fairly, we used a quantile-based stratified split instead of a pure random splitting. The data was partitioned into training (80%), validation (10%), and test (10%) sets to ensure that the pIC_50_ values were divided into equal-sized bins (quantiles), and samples from each bin were proportionally distributed into the training, validation, and test sets preventing the bias often associated with simple random sampling (Fig. 1).

### 2.2 Molecular descriptors calculation

Molecular descriptors are mathematically encoded quantitative representations of a molecule's chemical properties that form numerical values, enabling their integration into machine learning algorithms. These fingerprints represent the variety and similarity across molecules by encapsulating chemical structures in binary or continuous values. Here, we utilised RDKit, an open-source toolkit for calculating molecular descriptors using SMILES notation.^42^ 208 features, such as molecular weight, number of valence electrons, and radial electrons, were computed to understand the relationship between molecular structures and biological activity.

### 2.3 ML model development

This study employed three supervised regression algorithms – Linear Regression (LR), Support Vector Regression (SVR) and Random Forest (RF) to predict the pIC_50_ biological activity of novel compounds and enable virtual screening of top candidates. Variance threshold (VT) feature selection was first applied using Scikit-learn (threshold = 0.1) to remove low-variance molecular descriptors that provide little discriminative power, reducing the dimensionality and mitigating overfitting, followed by a pairwise Pearson correlation analysis where one feature from each pair having a correlation coefficient ≥0.90 was removed. We evaluated several established methods, including PCA, L1-based embedded selection (Lasso regularisation), and Recursive Feature Elimination (RFE), alongside the VT + correlation approach. While these advanced methods were tested, they introduced additional complexity and reduced the model's interpretability, as PCA converts physical descriptors into abstract components and L1/RFE-based selections depend heavily on algorithmic tuning. The VT + correlation framework, in contrast, provided a transparent and reproducible descriptor subset that effectively balanced simplicity, diversity, and predictive performance.

LR served as a baseline model, estimating pIC_50_ as a linear combination of selected descriptors. Its interpretability and simplicity make it a valuable benchmark in cheminformatics. The SVM regression was used to map features with kernel functions into high-dimensional spaces, enabling it to capture non-linear relationships between molecular descriptors and bioactivity. They are widely used in QSAR modelling, such as an SVM-based ensemble predicted potent HMG-CoA reductase inhibitors with high accuracy.^43^ The RF model was used to build an ensemble of decision trees trained on randomised subsets of descriptors and samples. It is effective at modelling complex descriptor–activity relationships while being robust to overfitting. RFs have demonstrated strong performance in various pIC_50_ prediction tasks, including FLT3 kinase inhibitors.^44^

### 2.4 ML model evaluation and virtual screening

After training and hyperparameter tuning of each model, their performance was assessed using mean absolute error (MAE), mean squared error (MSE), Pearson correlation (*R*) and *R*^2^. The model with the best predictive metrics was then applied to screen compound libraries for new potential inhibitors. The RF model was finally chosen for virtual screening as it performed the best on the training and test datasets. To navigate the extensive chemical space and identify promising compounds, public database libraries such as CHEMBL FDA APPROVED DRUGS, ZINC FDA APPROVED DRUGS and natural compound databases such as INDOFINENP, SPECSNP, IBSNP, and ACDISNP were employed against the RF model. Compounds with predicted pIC_50_ ≥ 7 were prioritised as potentially high-potent inhibitors. From these, we selected five molecules for further testing. These identified candidates were used for *in silico* and *in vitro* validation experiments.

### 2.5 Molecular docking

Genetic optimisation for ligand docking (GOLD) was used in the molecular docking study to gain a thorough knowledge of the binding mechanism of the potential drug compounds.^45^ GOLD uses a fitness score algorithm to assess and rank different binding modalities. The four main components of this function are the intramolecular hydrogen-bond contribution of the ligand, the intramolecular strain contribution inside the ligand, the hydrogen-bonding score between the protein and ligand, and the van der Waals interaction score between the protein and ligand. Furthermore, GOLD uses a genetic algorithm as a search algorithm to look into possible binding conformations and fitting sites to locate the ligand precisely in the binding site. In the present study, we used the default parameters of GOLD with the ChemPLP scoring function.

The structures of standard inhibitors, olutasidenib and α-mangostin, inactive inhibitor, temozolomide and screened inhibitors were downloaded from PubChem.^46^ For docking analysis, the X-ray crystallographic structure for mutant IDH1 complexed with olutasidenib (PDB ID: 6U4J), a known MT-IDH1 inhibitor was obtained from the Protein Data Bank.^47^ All water molecules, ligands and other non-standard hetero atoms were excluded from the protein structure including chains C and D. Hydrogen atoms were added to the crystal structure. 3D geometry optimisation of the ligand molecules was done by the Schrodinger LigPrep module (Schrödinger Release 2025-4: LigPrep, Schrödinger, LLC, New York, NY, 2025). The docking site was specified by a sphere at the geometric centre of the native ligand present in the crystal structure. For each independent algorithm, 3.5 Å maximum distance was set between hydrogen donors and fitting points. 4.0 Å cutoff was set for non-bonded van der Waals energies. The fittest pose was subsequently taken for a molecular dynamics (MD) simulation study.

### 2.6 MD simulation

MD studies were done to gain insight into the dynamic stability of ligand molecules within the protein binding site. This study utilised the GROMACS 2021.2 program.^48^ CHARMM GUI was used to pre-process the protein molecule. CHARMM General Force Field (CGenFF) server was used to build ligand topologies and CHARMM36 force field was employed during MD simulations.^49^

LINCS algorithm was used to constrain hydrogen bonds. Particle Mesh Ewald method was used for electrostatic interactions with a cutoff distance of 1.2 nm, and non-bonded interactions with a 10 Å cutoff for calculations. The temperature of the system was maintained at 298 K with a V-rescale thermostat, while a Berendsen barostat was used to keep pressure constant at 1 bar. The systems were equilibrated through 2 ns of NVT and 2 ns of NPT simulations prior to the production run. The production simulation was run for 250 ns, with snapshots saved every 100 ps.

### 2.7 Binding free energy analysis and per-residue decomposition studies

The binding free energy between protein-ligand complexes was calculated for all the systems using the molecular mechanics/Poisson–Boltzmann surface area (MM/PBSA) method.^50^ This tool utilises the system's internal energy, electrostatic and non-polar contribution to entropic and solvation contribution, combining molecular mechanics and continuum solvent models to compute free energy. The following equation can used for the MM/PBSA approach, that calculates the binding free energy (Δ*G*_bind_) between a protein and a ligand:

1 Δ*G*_bind_ = Δ*G*_MM_ + Δ*G*_PB_ + Δ*G*_SA_ − *T*Δ*S*

Here, the total gas-phase energy (sum of the Δ*E*_internal_ + Δ*E*_electrostatic_ + Δ*E*_vdw_) on the binding of interaction energy is shown as Δ*G*_MM_, between protein and ligand, the free energy of solvation is Δ*G*_SA_. Δ*G*_PB_ and Δ*G*_SA_ represent polar and nonpolar solvation energies, respectively. 1000 snapshots from each simulated trajectory of 250 ns were extracted from 150 ns to 250 ns. In the present study, entropy contributions were not included in the MM/PBSA calculations. Nonetheless, the MM/PBSA approach remains a robust and efficient method for extracting qualitative insights into protein–inhibitor interactions. In this work, the primary objective was to perform a comparative evaluation of the binding affinities of the selected lead molecules and to identify the key residues contributing to the enthalpic component of binding. The method reliably captures these relative trends and highlights the common and energetically significant residues involved in ligand stabilization at the MT-IDH1 binding site. The electrostatic, van der Waals and polar solvation energy components were computed using Adaptive Poisson–Boltzmann Solver (APBS). Non-polar energy contributions were estimated using Solvent-accessible surface area (SASA) with grid spacing and probe radius set to a value of 0.5 Å and 1.4 Å respectively. The dielectric constant was defined as 80 for the solvent, and 2 for the solute.

Per-residue decomposition analysis was done using the *g_mmpbsa* tool to gain measurable description of the energetic contribution for each amino acid with the inhibitors considered in the study.^50^ This tool decomposes the overall binding energy of the protein–ligand complex. Python scripts “*MmPbSaStat.py*” and “*MmPbSaDecomp.py*” were employed for MM/PBSA calculations and individual contributions of different amino acids.

### 2.8 Materials

Substrates, ICT (#42615) and NADP (#55615) for wild-type IDH1 enzyme assay were purchased from SRL. α-KG (#ASK1593) was procured from Avra, and NADPH (#99197) was acquired from SRL for MT-IDH1 enzyme assay. The stock solutions for each substrate were prepared in IDH1 assay buffer (20 mM Tris pH = 8.0, 10 mM NaCl, 10 mM MgCl_2_, 0.05% BSA). The standard IDH1 inhibitors, olutasidenib (#HY-114226), α-mangostin (#HY-N0328), and potential lead molecules, dacomitinib (#HY-13272), duvelisib (#HY-17044), idelalisib (#HY-13026), and vandetanib (#HY-10260), were purchased from MedChem Express. 10 mM DMSO stock solutions were prepared for each inhibitor and used for *in vitro* enzymatic assays.

### 2.9 Protein expression and purification

Human IDH1 (#S015804-01-A244736) and IDH1 R132H (#S015803-01-A244637) cloned into the pET41a (+) vector were purchased from Synbio Technologies, NJ, USA. The recombinant protein was expressed in BL21(DE3) by adding 1 mM IPTG for 6 hours at 4 °C in a bioreactor. Cells were resuspended in lysis buffer with a final concentration of 20 mM Tris (pH 8.0), 10 mM NaCl and passed through a homogeniser for efficient lysis. The lysate was then loaded onto a Ni-NTA column. After washing with 10 column volumes of wash buffer, the protein was eluted with elution buffer from a 0–100% gradient using ÄKTA Pure (Cytiva Life Sciences). The primary peak was collected and concentrated, corresponding to IDH1 and IDH1 R132H. Protein purity for both was assessed by SDS-PAGE. The protein estimation was done using the Bradford method, and concentrations were determined to be 4.07 and 4.37 mg mL^−1^, respectively (unpublished data). The purified enzymes were used for *in vitro* enzyme-based inhibition assays for standard and screened compounds.

### 2.10 Recombinant IDH1, and IDH1 R132H enzymatic assays

The IDH1 and IDH1 R132H biochemical assays were performed as previously described.^22^ Generally, WT IDH1 utilises NADP^+^ as a cofactor to catalyse the conversion of ICT to α-KG, while MT IDH1 utilises NADPH to reduce α-KG to 2-HG. The inhibitory potential of the compounds against IDH1 and IDH1 R132H can be determined by the detection of NADPH production or consumption. In a 1 mL reaction mixture, 880 µl assay buffer (20 mM Tris pH = 8.0, 10 mM NaCl, 10 mM MgCl_2_, 0.05% BSA), 20 µl α-KG substrate and 80 µl NADPH solution (at final concentration 1 mM and 100 µM, respectively) were added. The mixture was then incubated at 25 °C for 10 min, followed by measuring the OD values at 340 nm in kinetic mode. Substrates, ICT and NADP were added at a final concentration of 100 µM each for IDH1 WT assays. The change in absorbance over time was recorded, and specific activity (U mg^−1^) was calculated for the linear range and plotted against the substrate concentration. 10 mM DMSO stocks for each inhibitor were prepared to test and screen the drugs. The desired inhibitor concentration was added to a 1 mL reaction mixture before incubation with the enzyme. Post incubation, the same steps were repeated, and percentage-specific activity was plotted to assess the screened inhibitors.

## 3 Results and discussion

### 3.1 Development of ML models

Our study presents a novel drug design approach that integrates machine learning methods and bioinformatics tools for evaluating and screening the potential of known compounds as MT-IDH1 inhibitors. The dataset from the ChEMBL database was used against RDKit, an open-source molecular descriptors calculator using the SMILES notation to construct three machine learning predictive models – LR, SVR and RF, to identify and screen MT-IDH1 inhibitors. The presented regression model can be used for further research on IDH1 inhibitors. Fig. 1 highlights the distribution of pIC_50_ across the dataset used for ML model development. To further understand the diversity, Murcko scaffold (SI Fig. S1) and plain ring system (SI Fig. S2) analysis for the dataset was performed which revealed unique core combinations with frequencies of occurrence.

The LR model assumed a linear relationship between molecular fingerprints and pIC_50_ using a straight line. It involved multiple independent variables that minimise the sum of squared differences between observed and predicted values. Coefficients show how much the dependent variable changes when the independent variable changes by one unit, and the error terms capture the unexplained variability in prediction. While the LR model trained on all features exhibited instability and failed catastrophically with poor generalization on the test set, the model utilising selected features showed better performance and served as a benchmark. Nevertheless, it failed to adequately capture more non-linear patterns within the data. Therefore, we further explored nonlinear models such as SVR and RF, which can capture complex descriptor–activity relationships and potentially yield better generalisation performance. The SVR model was used to learn a nonlinear mapping from molecular descriptors to pIC_50_ values by fitting a regression hyperplane in the high-dimensional feature space. The RF algorithm, comprising multiple decision trees, each constructed randomly from the training set data, was selected and combined with their outputs to develop a more robust model.

Although the RF model trained on the full feature set achieved a marginally higher test *R*^2^ (∼0.68) compared with the feature-selected RF (∼0.66), we prioritized the feature-selected model. The reduction in dimensionality removes redundancy and multicollinearity, resulting in a more efficient, stable, and interpretable model without any meaningful loss in predictive accuracy. This decision was further supported by the behavior of the linear regression model. With all descriptors, the model achieved reasonable fits on the training and validation sets (*R*^2^_train = 0.648, *R*^2^_val = 0.423) but collapsed on the test set (*R*^2^_test = −7756.5), indicating extreme instability and overfitting. In contrast, after VT + correlation-based feature selection, its performance became stable and plausible across all splits (*R*^2^_train = 0.568, *R*^2^_val = 0.388, *R*^2^_test = 0.392), behaving as expected for a well-specified linear model. This contrast highlights how a highly collinear descriptor space can distort simpler models and mask genuine structure–activity relationships.

Hyperparameters for both algorithms were tuned systematically. For RF, we explored variations in the number of trees, maximum depth, maximum features per split, and minimum leaf size. The final configuration consisted of 600 trees, no depth limit, max_features = 0.5, and min_samples_leaf = 1. For SVR, we evaluated RBF kernels across a broad grid of *C*, *ε*, and *γ*, and the optimal model (*C* = 10, *ε* = 0.3, *γ* = 0.01) performed competitively but remained consistently inferior to RF on the test set. Taken together, these findings justify the selection of the feature-filtered RF model as the final model balancing predictive performance with improved robustness, interpretability, and reduced risk of variance inflation due to redundant or collinear descriptors. Summary of the performance metrics for each developed model is shown in Table 1.

**Table 1:** Performance and statistical evaluation of different machine learning models

Model	Dataset	MAE	MSE	*R* ^2^	Pearsons' *R*
RF (all features)	Train	0.191	0.069	0.944	0.9794
	Test	0.456	0.374	0.679	0.8276
**RF (selected features)**	**Train**	**0.194**	**0.069**	**0.944**	**0.9798**
	**Test**	**0.472**	**0.394**	**0.663**	**0.8165**
SVR (selected features)	Train	0.272	0.116	0.906	0.9538
	Test	0.475	0.413	0.646	0.8067
Linear regression (selected features)	Train	0.555	0.532	0.568	0.7535
	Test	0.647	0.710	0.392	0.6424

### 3.2 5-Fold cross validation and model generalization

To further validate generalisability, we performed 5-fold cross-validation (CV) on the training data for the best-performing models (RF and SVR) using the final VT + corr feature subset (98 descriptors). The mean cross-validated *R*^2^ values were 0.55 ± 0.04 for the RF model and 0.55 ± 0.05 for the SVR model, demonstrating highly consistent predictive behaviour across folds. The test-set *R*^2^ values (∼0.66–0.68) were slightly higher than the cross-validation averages. This difference was expected given the small dataset size and the inherent variability introduced by random sampling, rather than indicative of overfitting. Cross-validation evaluated performance across multiple randomised partitions, while the test-set score reflected the outcome of a single stratified split, which may occasionally align more favourably with the learned patterns. The close alignment between CV and test results indicated that both models exhibit stable, generalizable performance within the available chemical space. Although the modest dataset size naturally limits statistical precision, the narrow CV standard deviations and the absence of inflated test performance support the conclusion that the models are not learning noise.

**Fig. 2:**
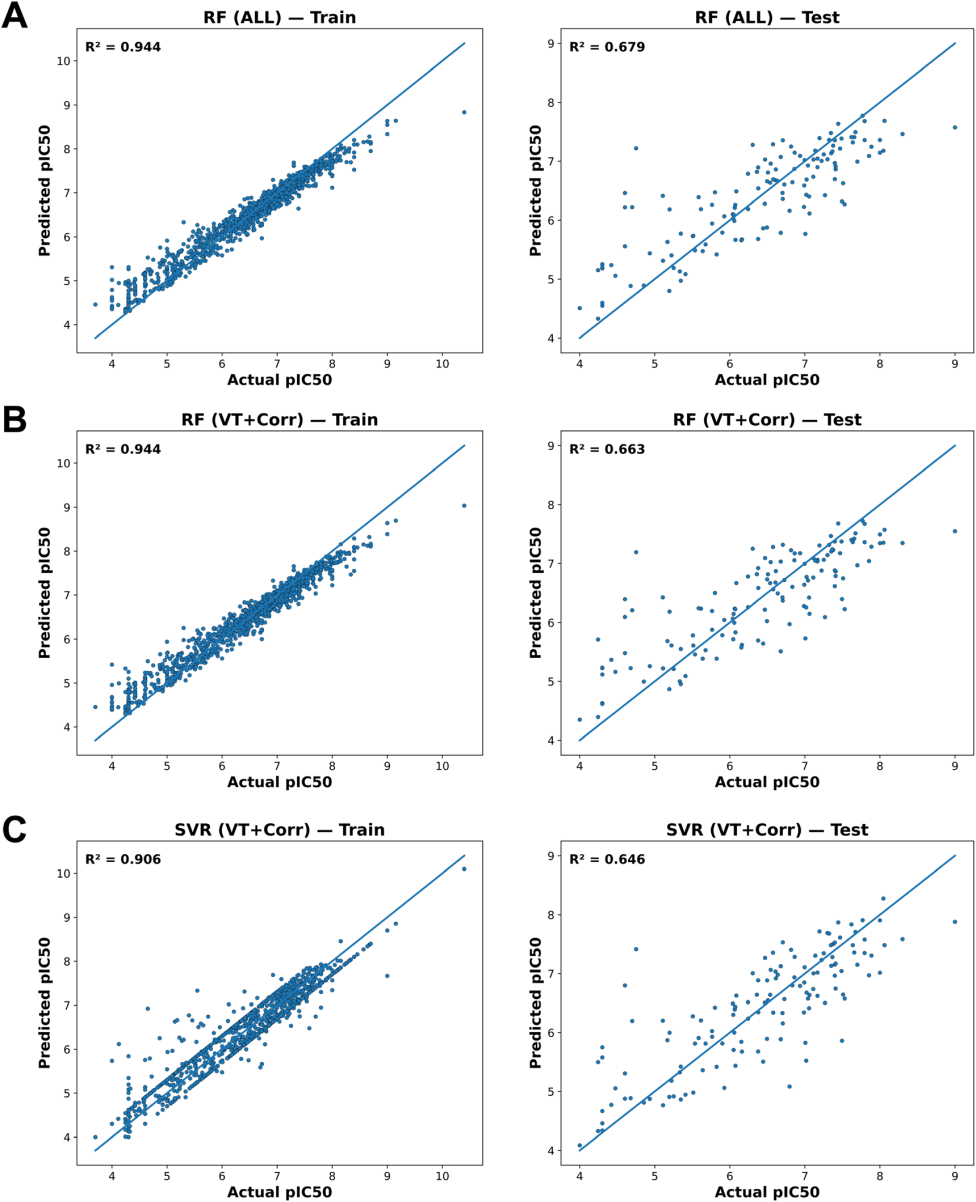
Scatter plots illustrating the predictive performance of machine learning models for pIC_50_ prediction. (A) Random Forest (RF) model trained using all available descriptors. (B) Random Forest model trained using a feature subset selected *via* variance threshold and correlation analysis (VT + Corr). (C) Support Vector Regression (SVR) model trained using the selected feature subset (VT + Corr). Each model is evaluated on both training (left column) and test sets (right column). The diagonal line denotes the ideal fit (*y* = *x*), where predicted values perfectly match actual values. Model performance is summarized by *R*^2^ values, with the Random Forest model demonstrating the highest predictive accuracy (*R*^2^ = 0.944 on training, 0.679 on test set) indicating superior generalizability and robustness of the Random Forest approach.

Overall, the combined cross-validation and external test results confirm that the final RF and SVR models generalize well and that their observed predictive performance is robust rather than an artifact of a particular dataset partition. Fig. 2 illustrates the correlation between actual and predicted values for both training and test datasets across SVR and RF models.

### 3.3 Feature selection and analysis

**Fig. 3:**
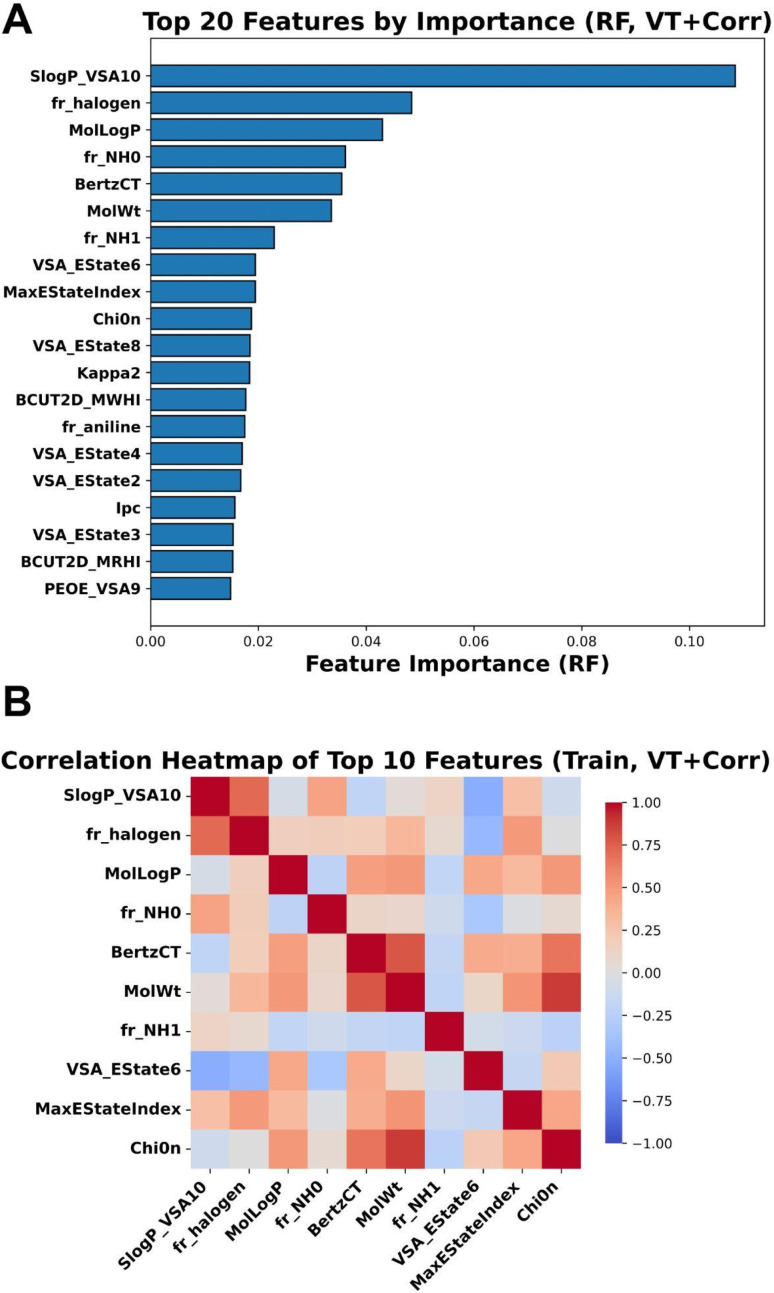
Feature importance and correlation analysis of molecular descriptors used in Random Forest modelling. (A) Bar plot showing the top 20 molecular features ranked by importance in the Random Forest model. The descriptor SlogP_VSA10 exhibits the highest predictive contribution, followed by fr_halogen and MolLogP, indicating a significant influence of hydrophobic surface patterns and halogenation on the model's predictions, (B) Pearson correlation heatmap of the top 10 most important features, illustrating the degree of linear correlation between them. Strong positive correlations are observed within the size/complexity cluster (*e.g.*, MolWt and BertzCT) are shaded in red, while negatively or weakly correlated pairs are shaded in blue, indicating potential redundancy or complementarity in model inputs.

The feature importance analysis was carried out to identify the contribution of each feature. The RF model is inherently robust to multicollinearity, and this analysis aids in interpretability and reinforces the relevance of both clustered and non-redundant features in driving the model performance. Among the top 20 features (Fig. 3A) critical in determining the model's accuracy, the SlogP_VSA10 type descriptor was the single most influential predictor in the model contributing over 10% to the total feature importance. This suggests that the hydrophobic distribution across the molecular surface plays a critical role in the bioactivity prediction. The log *P* value is derived from hydrophobic atomic constants that measures lipophilic contribution of individual atoms within a molecule, each described by adjacent atoms. Global physicochemical properties, specifically lipophilicity (MolLogP) and molecular size (MolWt), were also ranked among the top five features. The high ranking of MolLogP reinforces the finding that hydrophobicity is a primary driver of activity. The other essential features identified through feature importance analysis were, BertzCT, VSA_EState4, and VSA_EState2. The presence of halogens (fr_halogen) was the second most important feature, indicating that halogen bonding or specific steric effects may enhance potency. Additionally, nitrogen-containing fragments (fr_NH0, fr_NH1) and topological complexity (BertzCT) were significant, highlighting the importance of hydrogen bond acceptors/donors and specific molecular scaffolding. While these features were identified as highly important, Random Forest can sometimes split importance among correlated variables.

To ensure that the selected features represent distinct chemical information and to assess potential collinearity, a correlation heatmap was constructed to assess the interdependence among the most predictive molecular descriptors (Fig. 3B) for the top 10 features ranked by importance. Several descriptors exhibit moderate positive correlations, especially between: MolLogP, SlogP_VSA10, and MolWt, BertzCT and MolWt, and MaxEStateIndex and Chi0n. No pair of descriptors was observed to show a correlation approaching unity, confirming that the variance thresholding and correlation filtering (VT + Corr) approach effectively removed severe redundancy. The analysis revealed that while SlogP_VSA10 and MolLogP both characterize hydrophobicity, they exhibit negligible correlation (*r* ∼0), indicating that the model successfully distinguishes between specific hydrophobic surface patches (SlogP_VSA10) and global lipophilicity (MolLogP). Additionally, a distinct cluster of strong positive correlations (*r* > 0.8) was observed among MolWt, BertzCT, and Chi0n, reflecting the intrinsic collinearity between molecular weight, topological complexity, and connectivity. Halogen content plays a critical and independent role, indicating that specific substitution patterns significantly influence the endpoint being predicted. The presence of both highly correlated and relatively independent descriptors indicates a balanced feature set capturing diverse chemical properties relevant to the biological activity.

Collectively, the ML model development pipeline demonstrated that the modelling workflow yielded a robust and chemically interpretable RF model. The model predicted well for the training set (SI File S3) with the close agreement between cross-validation and external test performance confirming strong generalisation. With this framework in place, we deployed the optimised model to virtually screen candidate molecules for potential activity against MT-IDH1.

### 3.4 Screening of potential IDH1-MT inhibitors from public libraries

The molecular databases, CHEMBL FDA APPROVED DRUGS, ZINC FDA APPROVED DRUGS and natural compound databases such as INDOFINENP, SPECSNP, IBSNP, and ACDISNP were employed against the RF model to screen and identify potential hit compounds against the MT-IDH1. The screening from the RF model identified five FDA-approved drugs as possible candidates targeting IDH1 as described below:

*Atogepant*: (PubChem CID: 72163100, molecular weight: 603.52 Da) is a small-molecule orally available agonist of calcitonin gene-related peptide (CGRP) used in preventive therapy for episodic migraine headaches.^51^

*Dacomitinib*: (PubChem CID: 11511120, molecular weight: 469.93 Da) is a highly selective irreversible small-molecule inhibitor designed as (2E)-N-16-4-(piperidin-1-yl)but-2-enamide and is used for treatment of non-small cell lung cancer with epidermal growth factor receptor (EGFR) exon 19 deletion or exon 21 L858R substitution.^52^ It is a second-generation tyrosine kinase inhibitor characterised by the irreversible binding *via* covalent bonding to the cysteine residues in the catalytic domains of the HER receptors at the ATP domain of the EGFR family.

*Duvelisib*: (PubChem CID: 50905713, molecular weight: 416.87 Da), also known as IPI-145 and INK-1197, is a potent, reversible small molecule inhibitor targeting phosphatidylinositol 3-kinase (PI3K) delta and gamma for treatment of relapsed or refractory chronic lymphocytic leukaemia (CLL) or small lymphocytic lymphoma (SLL).^53^ The delta isoform controls cell proliferation and survival, whereas the gamma isoform is critical in cytokine signalling and proinflammatory response.

*Idelalisib*: (PubChem CID: 11625818, molecular weight: 415.43 Da) is an antineoplastic kinase inhibitor used for treating lymphocytic leukaemia (CLL), relapsed follicular B-cell non-Hodgkin lymphoma (FL), and relapsed small lymphocytic lymphoma (SLL) patients.^54^ Idelalisib targets the delta isoform (P110δ) of PI3K enzyme and induces the apoptosis of cancer cells and inhibit several signal transduction pathways, including B-cell receptor (BCR) signalling, and C-X-C chemokine receptor signalling.

*Vandetanib*: (PubChem CID: 3081361, molecular weight: 475.35 Da) is an orally available small-molecule antineoplastic kinase inhibitor of angiogenesis and cancer cell proliferation for the treatment of progressive medullary thyroid cancer. It is currently approved as an alternative to local therapies for unresectable and disseminated diseases. The mechanism of action involves selective inhibition of vascular endothelial growth factor receptor (VEGFR) and EGFR, which are altered during Transfection (RET) tyrosine kinases.^55^

All these screened molecules were studied for molecular docking, simulation and free energy studies to understand the molecular interactions and contributions in MT-IDH1 inhibition. Olutasidenib (PubChem CID: 118955396) and α-mangostin (PubChem CID: 5281650) were used as positive MT-IDH1 reference inhibitors. Temozolomide (PubChem CID: 5394) a chemotherapy drug was used as an inactive inhibitor or negative control for cross-validation and comparative analysis for *in silico* study.

### 3.5 Molecular docking analysis

**Fig. 4:**
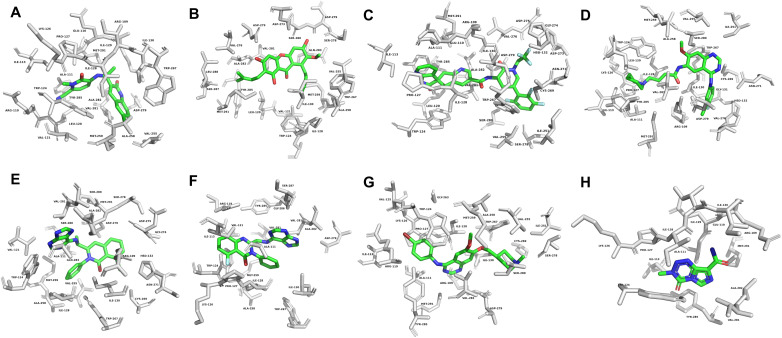
3D representation of molecular docked poses showing the binding site residues (up to 4 Å) in mutant IDH1 allosteric inhibitor binding site (A) olutasidenib, (B) α-mangostin, (C) atogepant, (D) dacomitinib, (E) duvelisib, (F) idelalisib, (G) vandetanib (H) temozolomide. Residues are shown as stick models, in green and ligand molecule as a default CPK colouring in stick. The protein backbone is displayed as a white cartoon.

The hit molecules were docked with the MT-IDH1 R132H protein (PDB ID: 6U4J) in the active site. Olutasidenib and α-mangostin are known IDH1 R132H inhibitors that are used as reference molecules in this study. The inhibitor olutasidenib (redock RMSD: 0.2301 Å) was used as a standard that could identify the near-native pose to compare and validate. Out of the poses generated, the best-established pose was selected based on docking and interaction tests. The results for molecular docking of five molecules obtained from the screening and three reference compounds are shown in Table 2. The 3D representation of molecular docked pose with the MT-IDH1 protein for each compound are presented in Fig. 4. The 2D interaction diagrams for each compound against MT-IDH1 depicting the interactions are presented in (SI Fig. S3).

**Table 2:** Docking score, important amino acids, and their individual contributions to ligand interactions for olutasidenib, α-mangostin, temozolomide and mutant IDH1-inhibitor screened complexes

Compound	ChemPLP score	Ligand interactions			
		Hydrogen bond	van der Waals	π–π	π–alkyl
Olutasidenib	109.99	Arg109, Leu120, Pro127, Ile128, Asp279	Arg119, Glu110, Val121, Lys126, Ile129, Ala282, Tyr285, Met291	Trp267	Ala111, Ile113, Trp124, Ile130, Val255, Ala258, Met259, Val281
α-Mangostin	63.59	Ser278, Asp279, Ser280	Leu120, Ile128, Ala258, Met259 Asp273, Asp279, Gln283, Ser287	—	Val121, Trp124, Ile130, Val255, Trp267, Val276, Val281, Ala282, Tyr285, Leu288, Met291
Atogepant	44.84	Arg109, Ala111, Cys269, Asp279, Ala282	Glu110, Leu120, Asp273, Gly274, Asp275, Val276, Ser278, Ser280, Met291	Trp267	Ile113, Trp124, Ile128, Pro127, Ile130, HSD132, Ile251, Val255, Val281, Tyr285
Dacomitinib	78.43	Arg109, HSD132, Ser280	Leu120, Pro127, Ile128, Lys126, Gly131, Ile251, Asn271, Asp279, Val281, Met291	Trp267	Ala111, Ile113, Trp124, Val255, Ala258, Met259, Val276, Tyr285
Duvelisib	71.45	HSD132, Asp279	Trp124, Val255, Asn271, Gly274, Asp275, Ser278, Ser280, Gln283, Met291	Trp267	Ala111, Val121, Ile128, Ile130, Ala258, Cys269, Val281, Ala282
Idelalisib	63.21	Pro127, Ile128, Ala282, Gly286	Ala111, Arg119, Ile130, Ala258, Met259, Trp267, Asp279, Val281, Tyr285, Ser287	—	Ile113, Val121, Trp124, Val281
Vandetanib	88.00	Pro127, Ile128, Ser278	Arg109, Arg119, Lys126, Ile130, Ile251, Val255, Gly263, Cys269, Asp279, Ser280, Tyr285	Trp124	Ala111, Ile113, Als258, Met259, Trp267, Val281
Temozolomide	45.41	Ile128	Arg109, Lys126, Ile129, Ile130, Ala282, Tyr285, Met291	Trp124	Ala111, Ile113, Pro127, Val281

Interaction analysis of protein–ligand complexes was done using Biovia Discovery Studio visualiser (v21.1.0, San Diego: Dassault Systèmes 2021) and PyMOL (The PyMOL Molecular Graphics System, Version 3.1.3, Schrödinger, LLC). The MT-IDH1 homodimer assumed the known open or inactive conformation, characterised by partially ordered α10 helix or the regulatory segment comprising residues from 271–286 in each monomer. In this state, the helix cannot coordinate the Mg^2+^ cofactor which is essential for enzymatic activity.^56^ The atomic contacts Leu120, Asp279, Ile128, Arg109, Trp124, and Val281 were identified as the most critical residues contributing to the stability of the olutasidenib and MT-IDH1 complex in the inhibitor binding pocket. These specific residues, such as those interacting with the inhibitor, contribute to MT-IDH1 selectivity.^57^ Temozolomide showed least interactions among the reference compounds. Table 2 highlights the summary of key interacting residues for olutasidenib, α-mangostin, temozolomide and the five screened molecules.

The potential screened drugs were also shown to form at least two hydrogen bonds in the binding pocket, with the drug stabilising each molecule. Atogepant formed three hydrogen bonds with the protein using Ser280, Asp279, and Cys269 and stabilised the complex using Trp267, Ile218, Ile130, Val281, Ala111, van der Waals and non-polar interactions. Dacomitinib, known to target catalytic domains of the HER receptors covalently, also efficiently targeted the MT-IDH1 protein through Ser278, Asp279, and Arg109 conventional hydrogen bond formation. At the same time, the significant non-polar interactions that contributed to its stability were provided by Trp267, Trp124, Ala111, Tyr285, Ala258, and Val281, significantly showing its potential to target MT-IDH1. Duvelisib, another promising screened inhibitor, showed significant non-polar contacts with Trp124, Leu120, Val281, Trp267, Ala258, and Ile130, contributing to its stability in the inhibitor binding pocket. Idelalisib and MT-IDH1 protein–ligand complex showed similar interaction profiles to other potential candidates and known IDH1 mutant inhibitors, olutasidenib and α-mangostin. Arg109 and Ile128 form conventional hydrogen bonds, with idelalisib contributing to its stability. Further structural stability was provided by non-polar contacts formed by residues Val281, Ile113, Arg119, Lys126, Tyr285, Trp124, and Ala282. Vandetanib was also shown to be a potential MT-IDH1 target. It forms conventional hydrogen bond interactions through Ile128 and Arg109, contributing to its inhibitor activity against MT-IDH1. It forms a carbon–hydrogen bond to the piperidine ring in vandetanib with Ser278. Further complex stability was provided by various non-polar atomic contacts contributed by Trp267, Val281, Ile130, Trp124, Ala111, Arg119, Ala282, and Tyr285.

Further, to enhance our understanding of the interactions and binding stability of all the screened potential candidate molecules in the active site of MT-IDH1, we performed molecular dynamics simulations for 250 ns. We computed the root-mean-square deviation (RMSD), radius of gyration (*R*_g_) and solvent-accessible surface area (SASA), which are valuable tools for evaluating the dynamics within the protein–ligand complexes.

### 3.6 MD simulation and MM/PBSA analysis

**Fig. 5:**
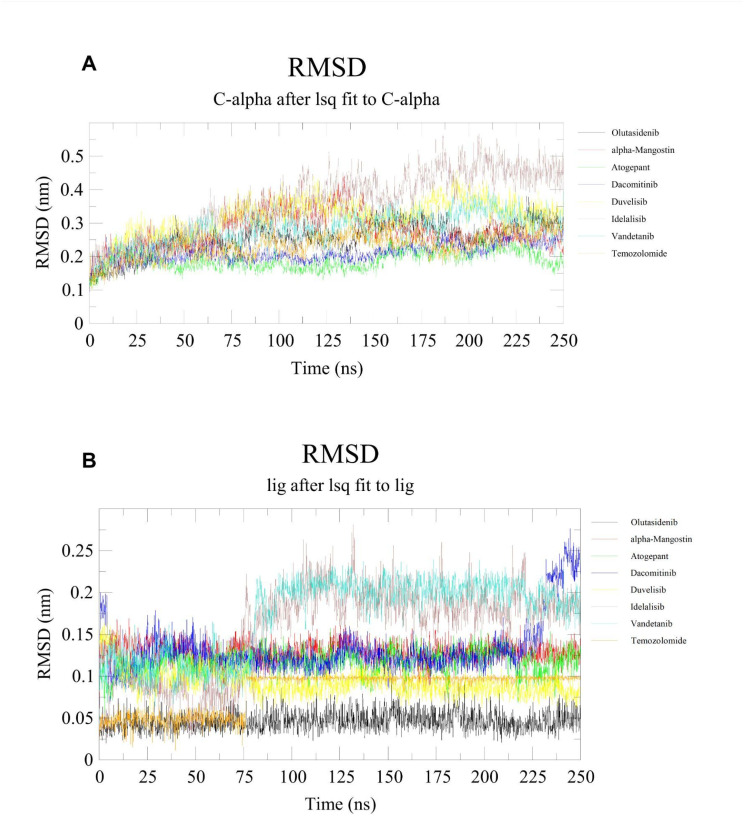
RMSD Plots showing structural dynamics upon binding with the inhibitors during 250 ns MD simulation (A) protein C-α, and (B) ligand, for standard mutant IDH1 inhibitors, olutasidenib (black) and α-mangostin (red), screened inhibitors, atogepant (green), dacomitinib (blue), duvelisib (yellow), idelalisib (brown), and vandetanib (turquoise), and inactive reference compound, temozolomide (orange).

**Fig. 6:**
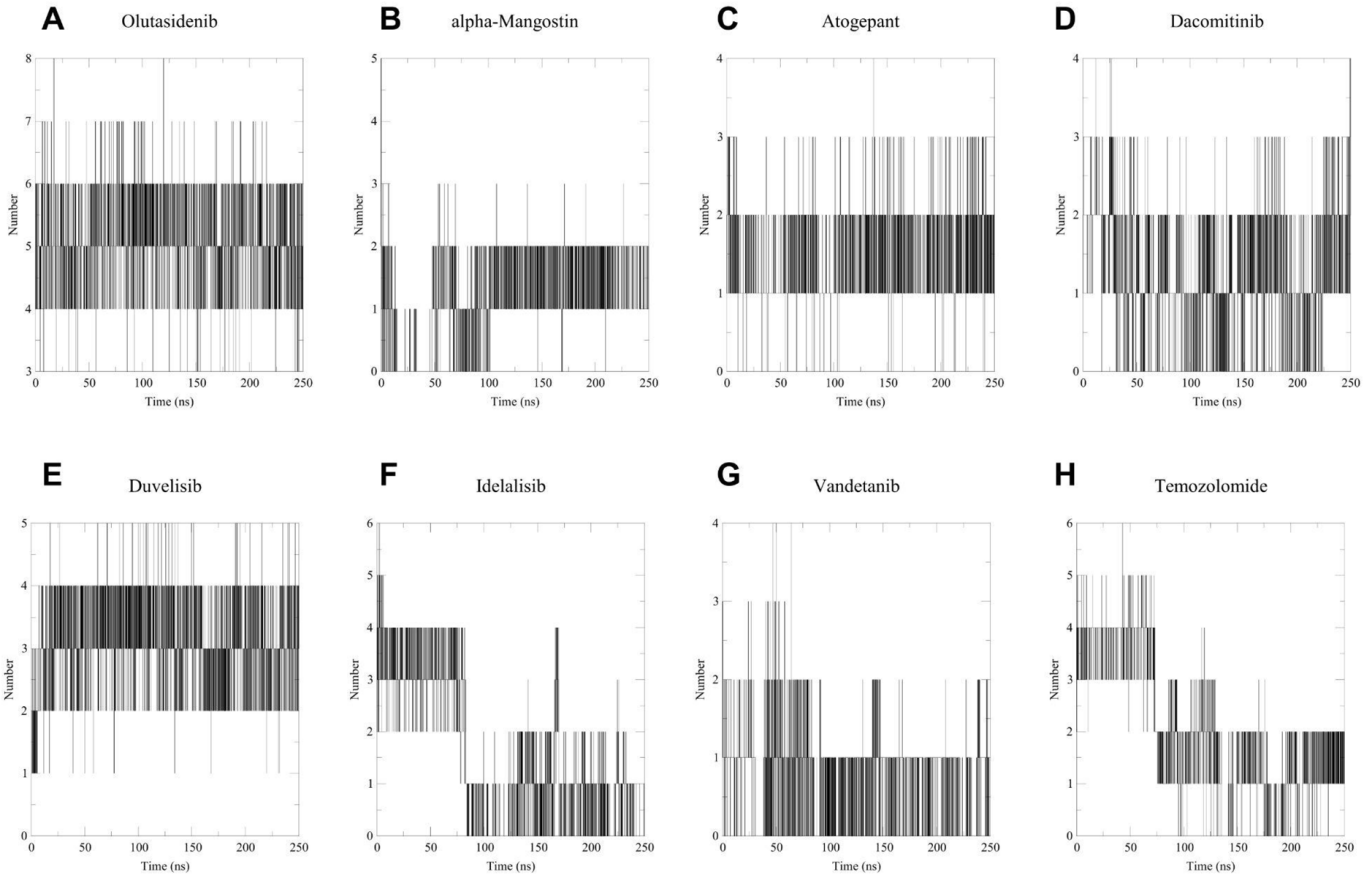
Hbond analysis during 250 ns MD simulation for standard mutant IDH1 inhibitors, (A) olutasidenib, (B) α-mangostin, screened inhibitors, (C) atogepant, (D) dacomitinib, (E) duvelisib, (F) idelalisib, and, (G) vandetanib, and inactive reference compound, (H) temozolomide. Each horizontal line on *y*-axis represents number of hydrogen bond formed simultaneously, with its duration shown along the time in (*x*) axis. A higher and denser clustering of lines suggests stronger and more stable interactions throughout the simulation period.

**Fig. 7:**
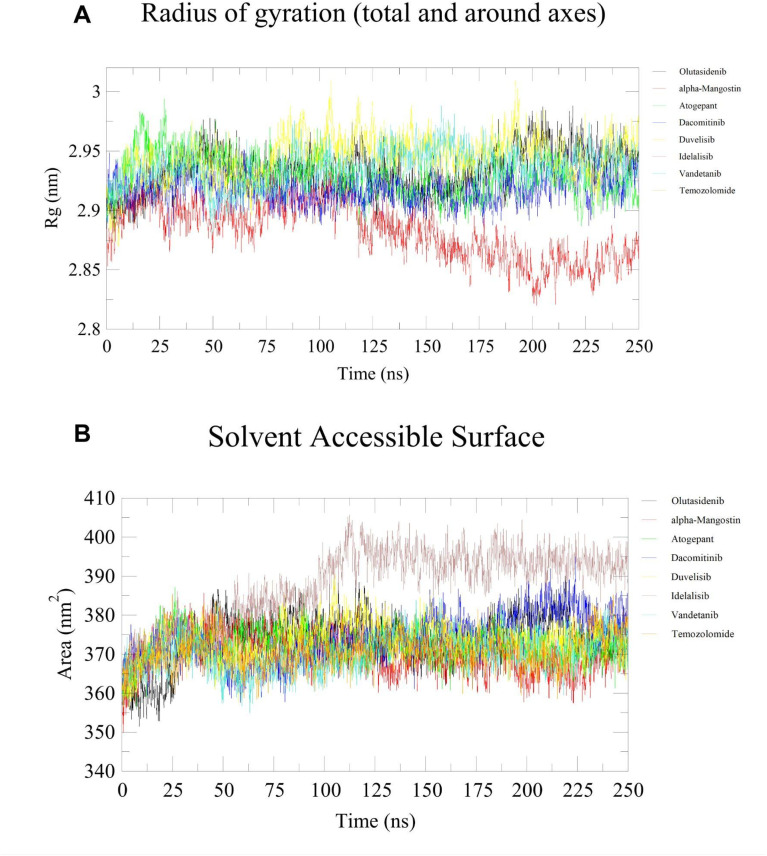
Plots illustrating post-MD (A) radius of gyration (*R*_g_) analysis, and (B) Solvent Accessible Surface Area (SASA) analysis over 250 ns simulation time for standard mutant IDH1 inhibitors, olutasidenib (black) and α-mangostin (red), screened inhibitors, atogepant (green), dacomitinib (blue), duvelisib (yellow), idelalisib (brown), and vandetanib (turquoise), and inactive reference compound, temozolomide (orange). Consistent *R*_g_ values indicate structural compactness and stability, whereas large fluctuations may reflect conformational flexibility or partial unfolding of the protein structure. An increase or fluctuation in SASA reflects changes in protein surface exposure to the solvent, which indicates conformational transitions or complex stability.

All screened inhibitors were studied to explore their atomic behaviour within a solvent environment using a 250 ns MD simulation for the best pose characterised by strong interactions and lowest binding energy conformation as derived from the molecular docking analysis. The 250 ns MD simulation was performed using GROMACS to check for the dynamic stability of these IDH1 inhibitor complex systems. To assess the conformational stability within the receptor binding pocket, protein Cα and ligand RMSD (Fig. 5) were measured with respect to the initial frame at 0 ns. The average protein Cα RMSD values of olutasidenib, α-mangostin, atogepant, dacomitinib, duvelisib, idelalisib, vandetanib and temozolomide were calculated to be 2.53 ± 0.47 Å, 2.74 ± 0.52 Å, 1.90 ± 0.28 Å, 2.11 ± 0.27 Å, 3.15 ± 0.56 Å, 3.63 ± 0.95 Å, 2.80 ± 0.49 Å, and 2.30 ± 0.38 Å respectively. These values exhibit a consistent trend throughout the study, indicating the stable behaviour of these inhibitor complexes. Unlike idelalisib, all other inhibitors appear to have attained stability early in the simulation and maintained general conformational stability throughout the simulation. Hbond analysis (Fig. 6) shows the number of hydrogen bond contacts formed by each inhibitor with the protein. Duvelisib was shown to form the highest and most stable hydrogen bond contact throughout the simulation followed by atogepant, and dacomitinib. However, other inhibitors also maintained at least two polar contacts throughout the simulation. Unlike the other compounds, temozolomide exhibited rapidly declining and largely unstable hydrogen-bond interactions across the simulation period, consistent with weak, non-specific binding to IDH1, supporting its known mechanism as a non-binding, indirect effector rather than a true IDH1 inhibitor. The *R*_g_ studies for all the screened molecules against the MT-IDH1 protein for 250 ns highlighted the degree of compactness of the structure. The overall value of *R*_g_ swings between 2.85 Å–3.05 Å, suggesting stable conformations throughout the simulation period, as shown in Fig. 7A. The graphical results for ligand property SASA were also computed to 250 ns, as illustrated in Fig. 7B. The relatively higher fluctuations in SASA and *R*_g_ were mainly observed for the idealisib–IDH1 complex. The increased fluctuations leading to inconsistent SASA and *R*_g_ originate primarily from the C-terminal region of the protein, which is inherently flexible. This behaviour was also visible in the RMSD plot and likely contributes to the observed variations.

**Fig. 8:**
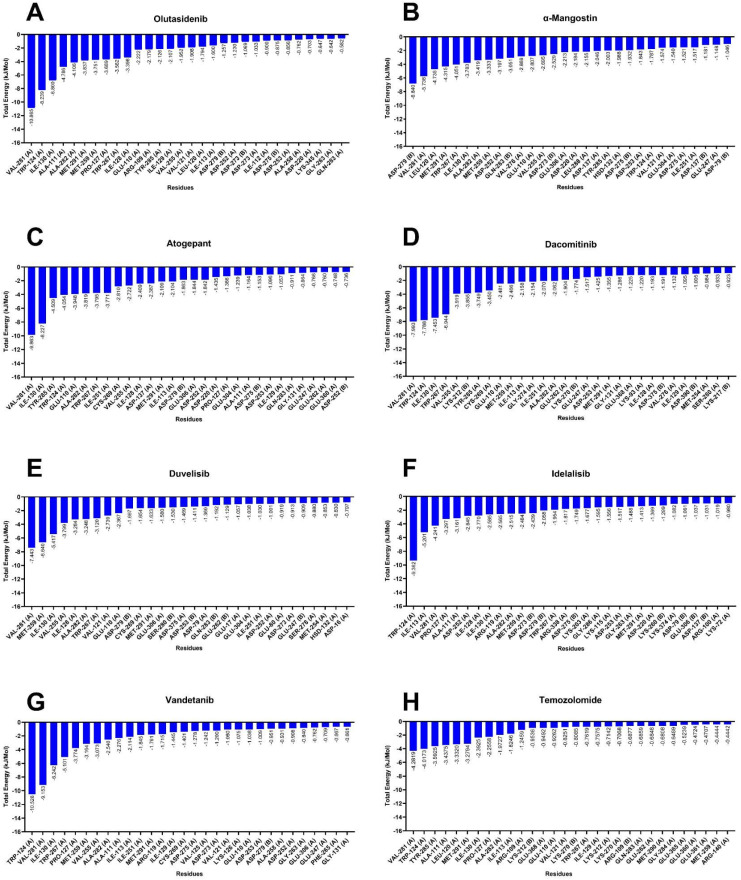
MM/PBSA graphs showing the top 30 per residue energy contributions for standard mutant IDH1 inhibitors, (A) olutasidenib and (B) α-mangostin, screened inhibitors, (C) atogepant, (D) dacomitinib, (E) duvelisib, (F) idelalisib, (G) vandetanib, and reference non-inhibitor (H) temozolomide. Residues on the *x*-axis are ranked based on their energetic contribution, with negative values on the *y*-axis indicating favourable interactions (*i.e.*, stronger binding affinity). Residues with the most negative energy values play key roles in stabilizing the ligand within the binding pocket, highlighting potential hotspots for ligand interaction and optimization.

We further employed the MM/PBSA to compute the binding free energy for all protein–ligand complexes. To access the prediction, 1000 frames were extracted from the final 100 ns segment (150 ns–250 ns) to calculate the MM/PBSA binding free energy along with standard deviation. These values are comparative enthalpic estimates that offers quantitative insights about the ligand binding potential within the inhibitor binding site. Fig. 8 represents the graphs of the top 30 per-residue contributions, and Table 3 summarises the MM/PBSA binding values for each molecule to the MT-IDH1. The positive reference compounds, olutasidenib and α-mangostin, had the highest binding free energy values of −123.67 kJ mol^−1^ and −119.71 kJ mol^−1^, respectively, suggesting and validating their highest affinity to the MT-IDH1 protein, while the negative non-inhibitor compound temozolomide showed weakest affinity score of −31.37 kJ mol^−1^ further affirming computational robustness. The relatively strong values of olutasidenib reflect the enthalpic nature of the calculation rather than an absolute experimental Δ*G*. As shown, strong van der Waals (−202.5 kJ mol^−1^) and electrostatic (−106.1 kJ mol^−1^) interactions, partially offset by polar solvation (+204.1 kJ mol^−1^), contribute to this total. This balance indicates that the strong value reflects the enthalpy-dominated nature of MM/PBSA calculations noting that while such methods can yield more negative values than experiment, the trend remains meaningful for comparative analysis. The MM/PBSA analysis of the screened inhibitors revealed their binding energies in the range of −70 kJ mol^−1^ to −80 kJ mol^−1^. These values suggest that there is a scope for lead optimisation, and these molecules have the potential to be an IDH1 target. Further, per-residue energy decomposition studies using MM/PBSA reveal the contribution of each amino acid residue in maintaining the stability of protein–ligand complexes. Val281, Trp124, Ile30, and Ala111 significantly contributed to the protein complexes' stability. Table 4 summarises the contribution of specific amino acids in sustaining the stability of the complexes in the inhibitor binding pocket of MT-IDH1.

**Table 3:** Summary of van der Waals, electrostatic, polar solvation, SASA and total binding energy (in KJ mol^−1^) for olutasidenib, α-mangostin, temozolomide and IDH1 inhibitors screened complexes calculated using MM/PBSA

Compound	Van der Waals energy	Electrostatic energy	Polar solvation energy	SASA energy	Binding energy
Olutasidenib	−202.513 ± 0.396	−106.120 ± 0.302	204.082 ± 0.283	−19.129 ± 0.023	−123.676 ± 0.360
α-Mangostin	−186.833 ± 0.397	−71.163 ± 0.285	160.700 ± 0.465	−22.423 ± 0.026	−119.711 ± 0.480
Atogepant	−254.511 ± 0.376	−64.192 ± 0.385	267.726 ± 0.486	−28.338 ± 0.028	−79.307 ± 0.467
Dacomitinib	−258.427 ± 0.382	−31.613 ± 0.379	244.844 ± 0.758	−26.768 ± 0.028	−71.981 ± 0.733
Duvelisib	−191.166 ± 0.385	−154.362 ± 0.395	301.626 ± 0.603	−20.715 ± 0.023	−64.607 ± 0.407
Idelalisib	−176.730 ± 0.332	−59.087 ± 0.514	180.921 ± 0.894	−21.010 ± 0.037	−75.888 ± 0.617
Vandetanib	−230.756 ± 0.375	−37.695 ± 0.380	215.822 ± 0.798	−23.998 ± 0.031	−76.608 ± 0.655
Temozolomide	−109.193 ± 0.251	−6.220 ± 0.311	96.264 ± 0.369	−12.200 ± 0.018	−31.372 ± 0.458

**Table 4:** Individual energy contribution of specific mutant IDH1 amino acid residues involved in the binding of olutasidenib, α-mangostin, temozolomide and mutant IDH1-inhibitor-screened complexes. (A) and (B) represents the protein chain. The values represent total energy contributions (in KJ mol^−1^) as calculated by MM/PBSA

Compound	Ala111	Leu120	Trp124	Ile130	Trp267	Asp279	Val281	X418 (inhibitor)
Olutasidenib	−4.7862	−1.7938	−8.2386	−6.8002	−3.5615	−1.2574 (B)	−10.8654	−61.068 ± 0.1878
α-Mangostin	−0.2708	−4.7349	−1.7869	−3.7828	−4.0508	−6.8398 (B)	−5.738	−73.445 ± 0.1794
Atogepant	−1.1642	−0.3025	−4.0537	−8.2272	−3.7948	−1.8633 (B)	−9.8632	−56.258 ± 0.2446
Dacomitinib	−0.2616	−0.614 (A)	−7.7881	−7.453	−6.9435	1.5275 (B)	−7.9932 (A)	−48.552 ± 0.2897
		−0.5075 (B)					−0.7338 (B)	
Duvelisib	−0.6687	−0.4661 (A)	−0.6307	−5.4168	−3.1199	−1.6969 (B)	−7.4434 (A)	−30.669 ± 0.2064
		−0.5953 (B)					−0.6598 (B)	
Idelalisib	−3.1605	0.031	−9.3619	−2.5848	−1.9535	−2.058 (B)	−4.241	−28.601 ± 0.3797
Vandetanib	−2.276	−0.3682 (B)	−10.5278	−6.2418	−5.1005	−0.9505 (B)	−9.1531	−32.779 ± 0.3037
Temozolomide	−3.4375	−3.3320	−4.0173	−2.3925	−0.7619	11.3993	−4.2819	−4.5138 ± 0.2435

### 3.7 Biochemical analysis of potential compounds

**Fig. 9:**
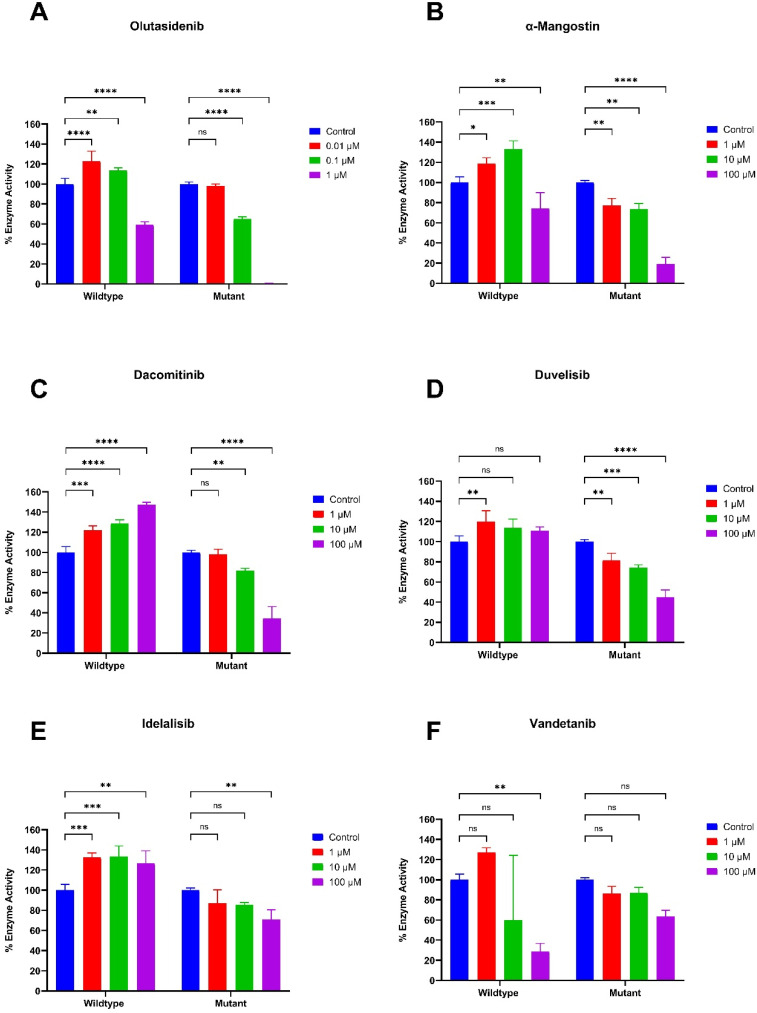
Comparative *in vitro* enzyme-based assay of standard inhibitors, (A) olutasidenib and (B) α-mangostin, and screened inhibitors, (C) dacomitinib, (D) duvelisib, (E) idelalisib, and (F) vandetanib, against IDH1 wild-type and mutant R132H. Enzymatic activity was quantified and normalised to untreated control (blue bars), and the results are expressed as percentage enzyme activity. All data are presented as mean ± SEM from triplicate experiments. Statistical analysis was performed using one-way ANOVA with significance *p < 0.05, **p < 0.01, ***p < 0.001, and ****p < 0.0001.

The spectrophotometry-based IDH1 biochemical assay was used to evaluate the ability of the screened candidates for inhibitory enzyme activity against IDH1 R132H. The upstream bioprocessing of recombinant wildtype and MT-IDH1 R132H enzyme was done using a bioreactor and purified using Ni-NTA column chromatography in AKTA Pure as described in the methodology. Protein quality was assessed by SDS-PAGE, and the optimal reaction conditions were determined, including *K*_m_ and *V*_max_ values for the substrates (unpublished data, SI Fig. S4 and S5). Similar to *in silico* studies, *in vitro* enzyme-based assay utilised olutasidenib, and α-mangostin as reference compounds for comparative analysis. Four of five screened compounds were used for further *in vitro* enzyme-based screening (Fig. 9).

Olutasidenib, a highly potent MT-IDH1 inhibitor, at 1 µM, the wildtype enzyme retains ∼60% activity but only ∼0.1% by the mutant, demonstrating strong mutant selectivity. While olutasidenib is a nanomolar inhibitor, our assay (Fig. 9A) demonstrated that complete inhibition of the mutant enzyme requires a concentration of 1 µM, with partial activity retained at 0.1 µM (100 nM). This establishes the dynamic range of our assay and confirms that sub-micromolar potency results in dose-dependent inhibition while retaining the IDH1 wild-type enzyme activity. α-mangostin, a natural compound obtained from dietary xanthose, known to inhibit MT-IDH1 was also used as another positive control. The IDH1 wildtype enzyme activity for α-mangostin did not decrease with changing concentration of the inhibitor. However, the activity gradually reduced for MT-IDH1 with increasing concentrations. At 100 µM, α-mangostin decreased the MT-IDH1 activity by 80%, in contrast to 23% by the wild-type IDH1. α-Mangostin exhibited a dose-dependent reduction in activity (Fig. 9B) in the micromolar range (1–100 µM), consistent with literature reports. These results confirm that the assay system is specific and capable of detecting inhibition at sub-micromolar concentrations.

The enzyme activity for screened compounds (Fig. 9C–F) in our study showed a significant reduction in MT-IDH1 enzyme activity compared to wild-type IDH1, suggesting their potential to be developed as potent MT-IDH1 therapeutics. Dacomitinib, was shown to inhibit MT-IDH1 with increasing concentration up to 100 µM, while acting as an agonist against wildtype IDH1. Duvelisib, a small-molecule inhibitor against PI3K, also exhibited a significant decrease in the enzyme activity of MT-IDH1 with increasing inhibitor concentration. At 100 µM concentration, the enzyme activity was reduced to 38% while there was no significant change in activity for wild-type IDH1, suggesting it as a potential candidate for IDH1 mutant therapy. Idelalisib, an antineoplastic kinase inhibitor used for treating CLL, also showed IDH1 inhibitory activity specific to MT-IDH1 compared to wild-type IDH1 in our assay. It reduced the specific activity by only 20% at 100 µM concentration, highlighting the need for further optimisation to develop a potent MT-IDH1 inhibitor for gliomas. Vandetanib, a classified orally available small molecule antineoplastic kinase inhibitor targeting VEGFR, did not show the expected results among all the molecules screened. It showed a significant decrease in IDH1 wildtype activity compared to insignificant changes in MT-IDH1 specific activity. The wildtype activity was reduced to 28% at 100 µM concentration, suggesting a strong binding affinity for wildtype compared to MT-IDH1. This finding also aligns with a recent study that proposed its therapeutic use in ACVR1-mutant diffuse intrinsic high-grade pontine glioma, a subtype typically lacking IDH1 mutations.^58^ The observed wildtype-specific inhibition may reflect its suitability in targeting metabolic pathways active in IDH1 wildtype high-grade gliomas, rather than MT-IDH1 lower-grade gliomas.

Although the screened inhibitors exhibited only micromolar inhibition compared to the higher potency predicted in computational studies, they do not reflect any issue with the specific binding. This discrepancy between the absolute calculated energies and the experimental IC_50_ values arises from methodological and physicochemical factors. Computational screening often overestimates inhibitor potency because it assumes rigid protein conformations, ideal ligand poses and minimal solvent or entropic effects.^59,60^*In vitro* assays, however, use recombinant IDH1 that displays natural flexibility, variable loop dynamics and differences in cofactor or post-translational state, all of which reduce effective binding. Moreover, olutasidenib is the product of extensive SAR-driven optimisation, whereas the compounds identified in our study are early-stage hits with repurposing potential, intended as starting points for future optimisation. Additionally, common biochemical factors, such as compound instability, aggregation in aqueous buffers, shifts in inhibitor protonation state, or nonspecific binding to assay components can shift the apparent inhibitory concentration into the micromolar range, without compromising specificity. Although the precise molecular mechanism by which these repurposed drugs inhibit IDH1-mutant enzyme requires further characterisation, their primary significance lies in their established pharmacological history. Given that these compounds were originally optimised for distinct therapeutic targets, their micromolar activity against IDH1 represents a promising ‘off-target’ potential suitable for repurposing. Collectively, the inhibitors displayed the expected kinetic behaviour typical of the MT-IDH1 mechanism, substantiating that the observed activity arises from specific and on-target enzyme inhibition rather than non-specific effects, while providing a solid foundation for their further development as targeted therapeutics.

## 4 Conclusions

In this study, we developed a machine learning-based algorithm that could predict the potency of the chemical compounds against IDH1. Among the three models trained, random forests outperformed with best accuracy and generalisability. Further, we screened a public library using computational approaches to study and identify potential compounds targeting MT-IDH1 R132H in gliomas. The screening identified five compounds, atogepant, dacomitinib, duvelisib, idelalisib, and vandetanib that are already known drug candidates for other targets. We tested their efficacy against MT-IDH1 R132H using *in silico* drug discovery approaches. The molecular docking, MD simulation and free energy MM/PBSA calculations validated their potency towards the MT-IDH1 target. Biochemical evaluations using *in vitro* enzyme-based assays provide direct evidence that three of the screened compounds disrupt MT-IDH1 function efficiently. These findings bridge the gap in predicting drug inhibitory activity while providing a robust ML model to facilitate the screening and development of potent IDH1-targeting therapeutics. The identified compounds hold promise as inhibitors of MT-IDH1 R132H with their target interaction suggesting a significant potential for further exploration. Future work will focus on detailed structural and functional analysis with chemical optimisation of of these scaffolds to enhance their efficacy and selectivity. Additional studies are essential to determine their potency, stability, and therapeutic potential. If successfully developed, these inhibitors could contribute to novel treatment strategies for malignancies driven by IDH1 mutations.

## Author contributions

Mayank Bajaj: writing original manuscript draft, visualisation, validation, investigation of *in silico* and *in vitro* methodology, formal analysis, and data curation. Tanneru Hemanth Kumar, Rohit Kumar, Vishal Pandey: development of AI-ML model, library screening and visualisation, Sahanawaz Parvez: review and editing manuscript, investigation of *in silico* analysis. Polamarasetty Aparoy: conceptualisation, review and editing manuscript, visualisation, methodology, investigation, data analysis, and supervision. Roy Karnati: conceptualisation, writing, review and editing manuscript, data analysis, supervision, project administration, and funding acquisition.

## Conflicts of interest

The authors declare that they have no known competing financial interests or personal relationships that could have appeared to influence the work reported in this paper.

## Note added after first publication

This article replaces the version published on 18 December 2025, which included an incorrect Data availability statement.

## Supplementary Material

RA-015-D5RA06290J-s001

RA-015-D5RA06290J-s002

RA-015-D5RA06290J-s003

RA-015-D5RA06290J-s004

## Data Availability

The chemical libraries analysed in this work were sourced from ChEMBL, ZINC, INDOFINENP, SPECSNP, IBSNP, and ACDISNP. The dataset of compounds used for model building, as well as data supporting the *in-vitro* enzyme assays, is included as part of the supplementary information (SI). See DOI: https://doi.org/10.1039/d5ra06290j. The relevant code, model scripts, and computational workflow presented in this article can be accessed using GitHub link at URL: https://github.com/rohit-iipeml/IDH.git.
