# Supplementary material for: Integrative machine learning-guided in silico and in vitro approach reveals selective small molecule inhibitors targeting mutant IDH1
Source: RSC Adv. 2025 Dec 18;15(59):50944–62. doi: 10.1039/d5ra06290j (PMC12713622; doi:10.1039/d5ra06290j)

Supplementary Information (SI) for RSC Advances.
This journal is © The Royal Society of Chemistry 2025

SUPPLEMENTARY DATA

Integrative Machine Learning-Guided *In-silico* and *In-vitro* Approach Reveals Selective Small Molecule Inhibitors Targeting Mutant IDH1

Mayank Bajaj[1], Rohit Kumar[2], Vishal Pandey[2], Shahnawaz Parvez[2], Tanneru Hemanth Kumar[3], Aparoy Polamarasetty*[2], Roy Karnati*[1]

[1]Translational Biology Laboratory, Department of Animal Biology, School of Life Sciences, University of Hyderabad, Hyderabad – 500046, Telangana, India

[2]Molecular Modelling and Protein Engineering Lab, Indian Institute of Petroleum and Energy, Visakhapatnam – 530003, Andhra Pradesh, India

[3]Department of Chemical Engineering, Indian Institute of Petroleum and Energy, Visakhapatnam – 530003, Andhra Pradesh, India

*Corresponding authors:

Dr. Roy Karnati, Associate Professor

Postal Address: Translational Biology Lab, Department of Animal Biology, School of Life Sciences, University of Hyderabad, Gachibowli – 500046, Hyderabad, Telangana

Email: roykarnati@uohyd.ac.in

Phone: +91-9652921092

Dr. Aparoy Polamarasetty, Associate Professor

Postal Address: Molecular Modelling and Protein Engineering Lab, Indian Institute of Petroleum and Energy, Visakhapatnam – 530003, Andhra Pradesh

Email: aparoy.bio@iipe.ac.in

Phone: +91-8219241123

Files

Supplementary File S1: Curated dataset of 1631 compounds used for model building and validation with descriptor data and pIC50 values

Supplementary File S2: Final dataset of 1342 structurally diverse compounds curated after applying Tanimoto similarity filter (< 0.85) randomly split in an 80:20 ratio into training and test sets.

Supplementary File S3: The actual and predicted pIC50 values of 1631 compounds using selected features on Random Forest model highlighting robustness and potential of this model for the prediction of biological activity of unknown compounds.

Figures

Supplementary Figure S1: The figure represents most frequent Murcko scaffolds identified for the ML dataset of bioactive compounds against IDH1. Each structure represents a distinct core framework, with the count indicating corresponding frequencies of occurrence. These scaffolds highlight key structural backbones and the recurring chemotypes within the dataset.

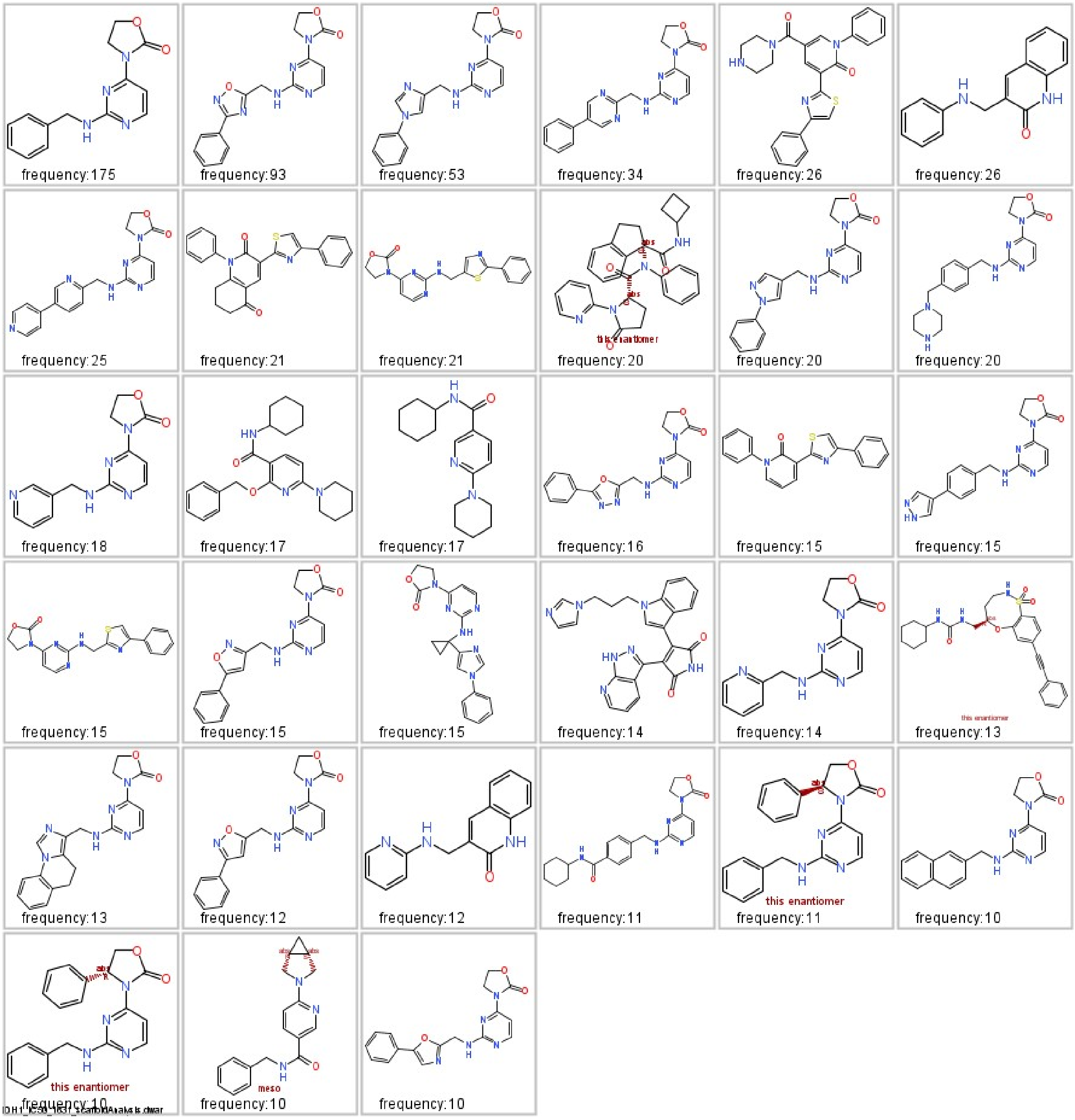

Supplementary Figure S2: This figure illustrates the most common plain ring scaffolds found in a dataset of bioactive compounds. Each structure represents a simple, unbranched ring system without side chains or complex substitutions. The associated frequency indicates how often each ring appears across the dataset. These core ring motifs form the foundational elements of many drug-like molecules and are essential for understanding recurring chemical patterns in medicinal chemistry.

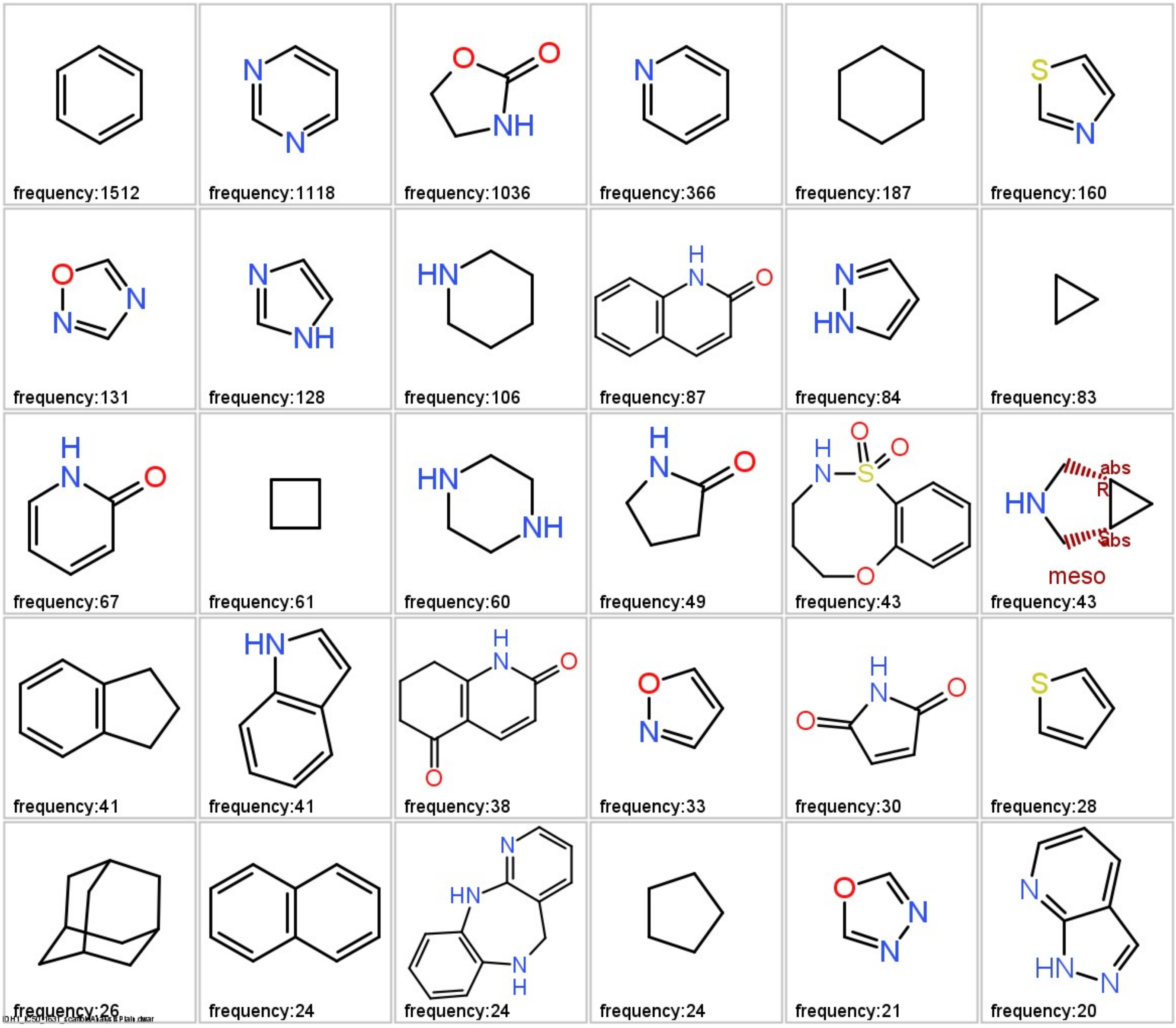

Supplementary Figure S3: The 2D interaction diagrams for each compound against MT-IDH1 depicting the interactions ***A)*** *Olutasidenib,* ***B)*** *α-Mangostin,* ***C)*** *Atogepant,* ***D)*** *Dacomitinib,* ***E)*** *Duvelisib,* ***F)*** *Idelalisib,* ***G)*** *Vandetanib* ***H)*** *Temozolomide.*

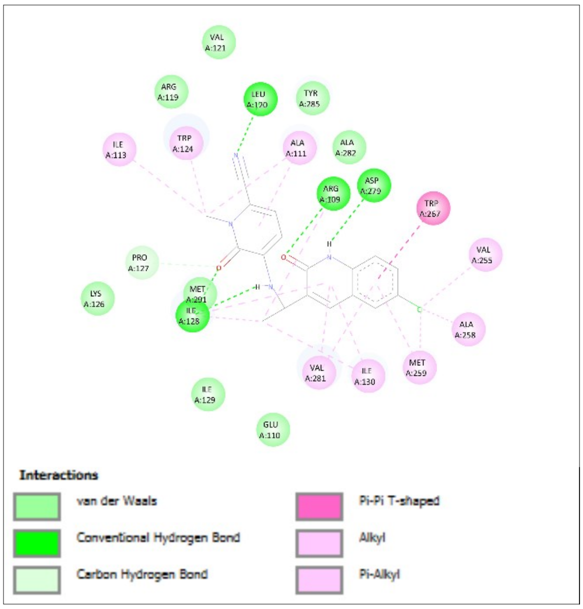

A)

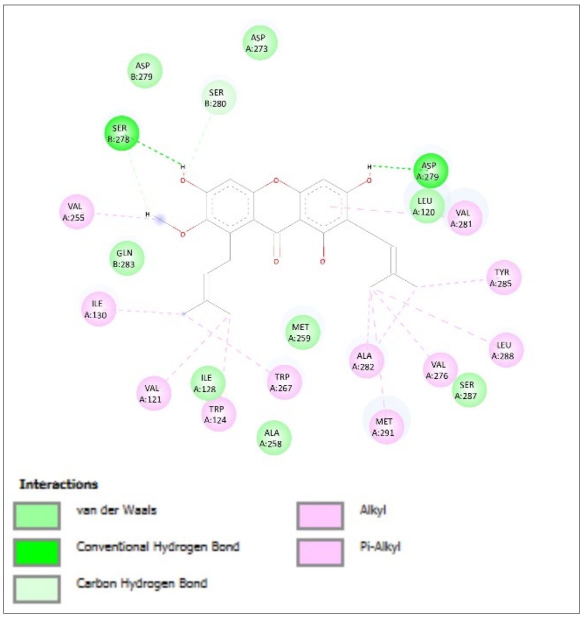

B)

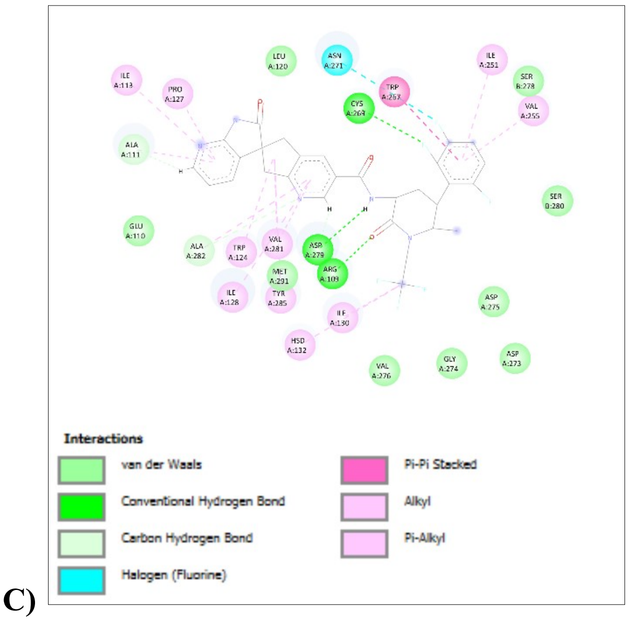

C)

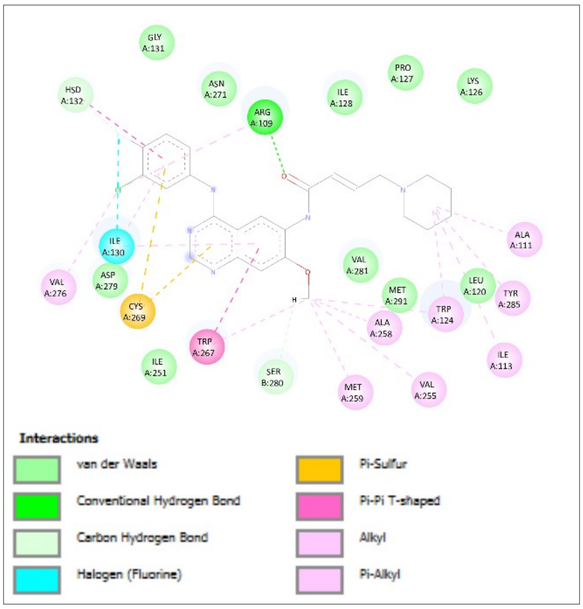

D)

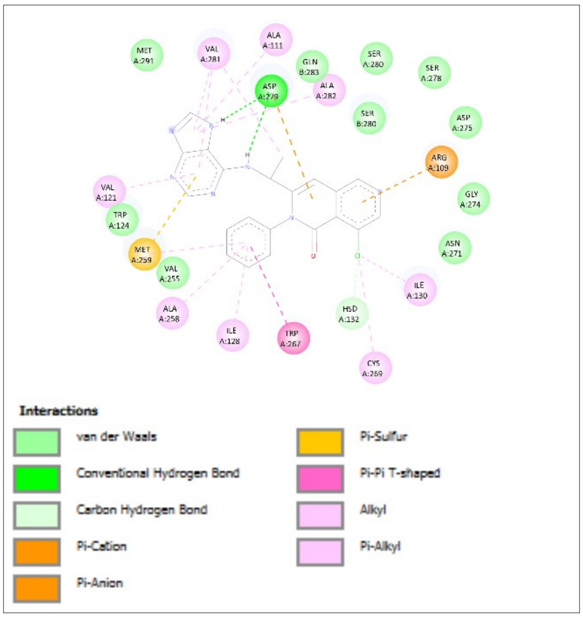
MET A:291
VAL A:281
ALA A:111
GLN B:283
SER A:280
SER A:278
ASP A:279
ALA A:282
SER B:280
ASP A:275
ARG A:109
GLY A:274
VAL A:121
TRP A:124
MET A:259
VAL A:255
ASN A:271
ILE A:130
ALA A:258
ILE A:128
TRP A:267
HSD A:132
CYS A:269
Interactions
van der Waals
Conventional Hydrogen Bond
Carbon Hydrogen Bond
Pi-Cation
Pi-Anion
Pi-Sulfur
Pi-Pi T-shaped
Alkyl
Pi-Alkyl
E)

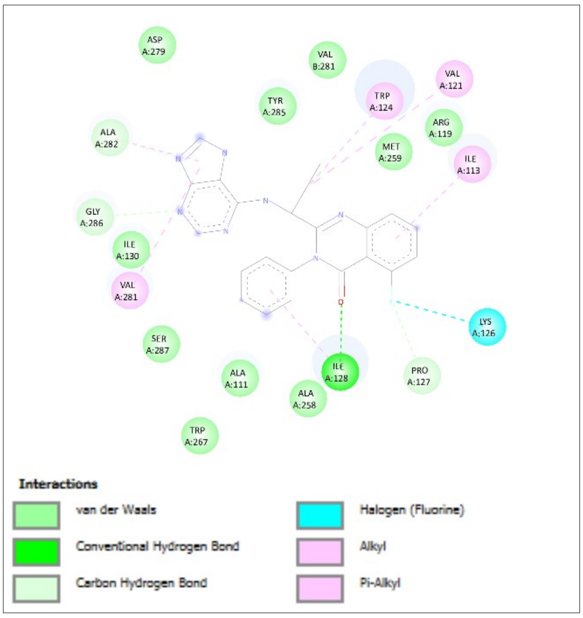
ASP A:279
VAL B:281
TYR A:285
TRP A:124
VAL A:121
ARG A:119
MET A:259
ILE A:113
ALA A:282
GLY A:286
ILE A:130
VAL A:281
SER A:287
ALA A:111
ILE A:128
ALA A:258
PRO A:127
LYS A:126
TRP A:267
Interactions
van der Waals
Conventional Hydrogen Bond
Carbon Hydrogen Bond
Halogen (Fluorine)
Alkyl
Pi-Alkyl
F)

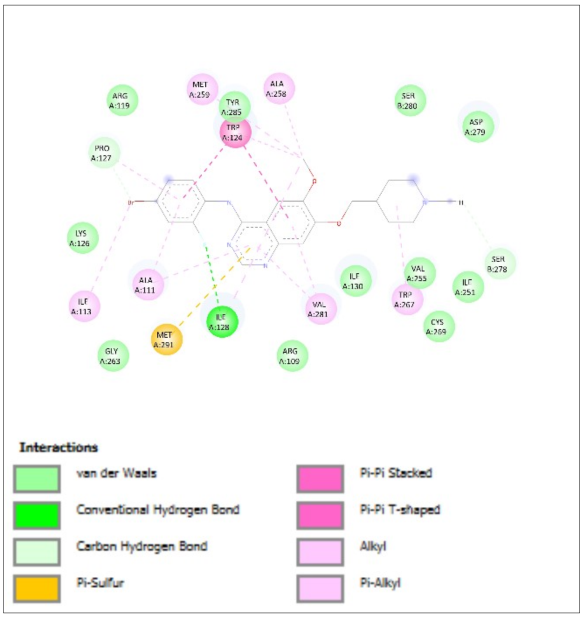
ARG A:119
MET A:259
TYR A:285
ALA A:258
TRP A:124
SER B:280
ASP A:279
PRO A:127
LYS A:126
ALA A:111
ILE A:113
ILE A:128
MET A:291
GLY A:263
VAL A:281
ILE A:130
ARG A:109
VAL A:255
TRP A:267
ILE A:251
SER B:278
CYS A:269
Interactions
van der Waals
Conventional Hydrogen Bond
Carbon Hydrogen Bond
Pi-Sulfur
Pi-Pi Stacked
Pi-Pi T-shaped
Alkyl
Pi-Alkyl
G)

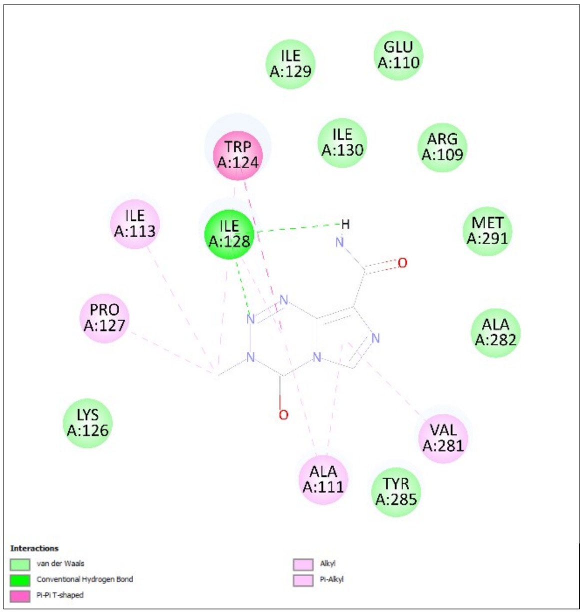
ILE A:129
GLU A:110
TRP A:124
ILE A:130
ARG A:109
ILE A:113
ILE A:128
MET A:291
PRO A:127
ALA A:282
LYS A:126
VAL A:281
ALA A:111
TYR A:285
Interactions
van der Waals
Conventional Hydrogen Bond
Pi-Pi T-shaped
Alkyl
Pi-Alkyl
H)

Supplementary Figure S4: SDS-PAGE analysis for recombinantly purified mutant IDH1.

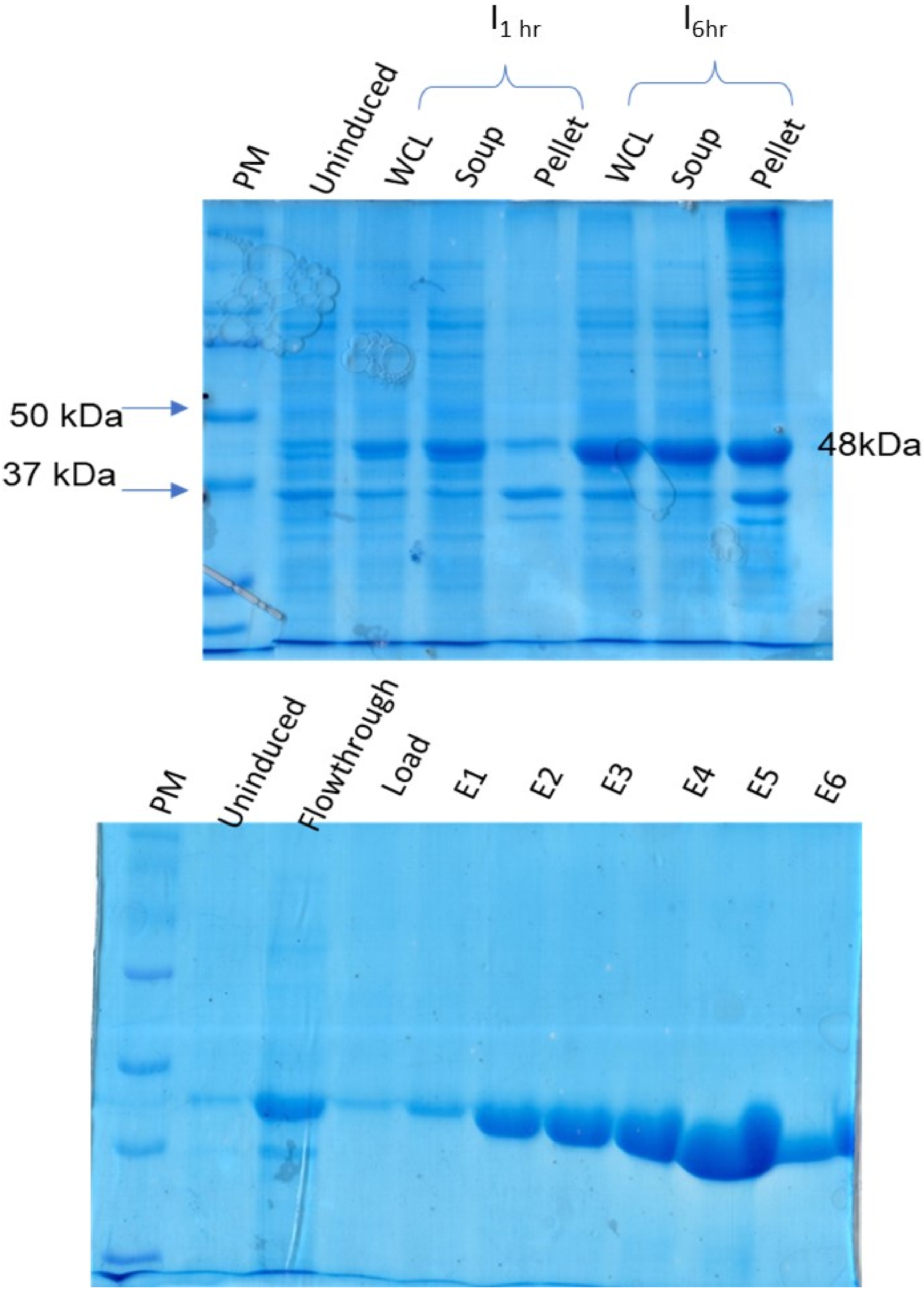

Supplementary Figure S5: Calculation of kinetic parameters, Km and Vmax for purified MT-IDH1 protein for α-KG and NADPH substrates.

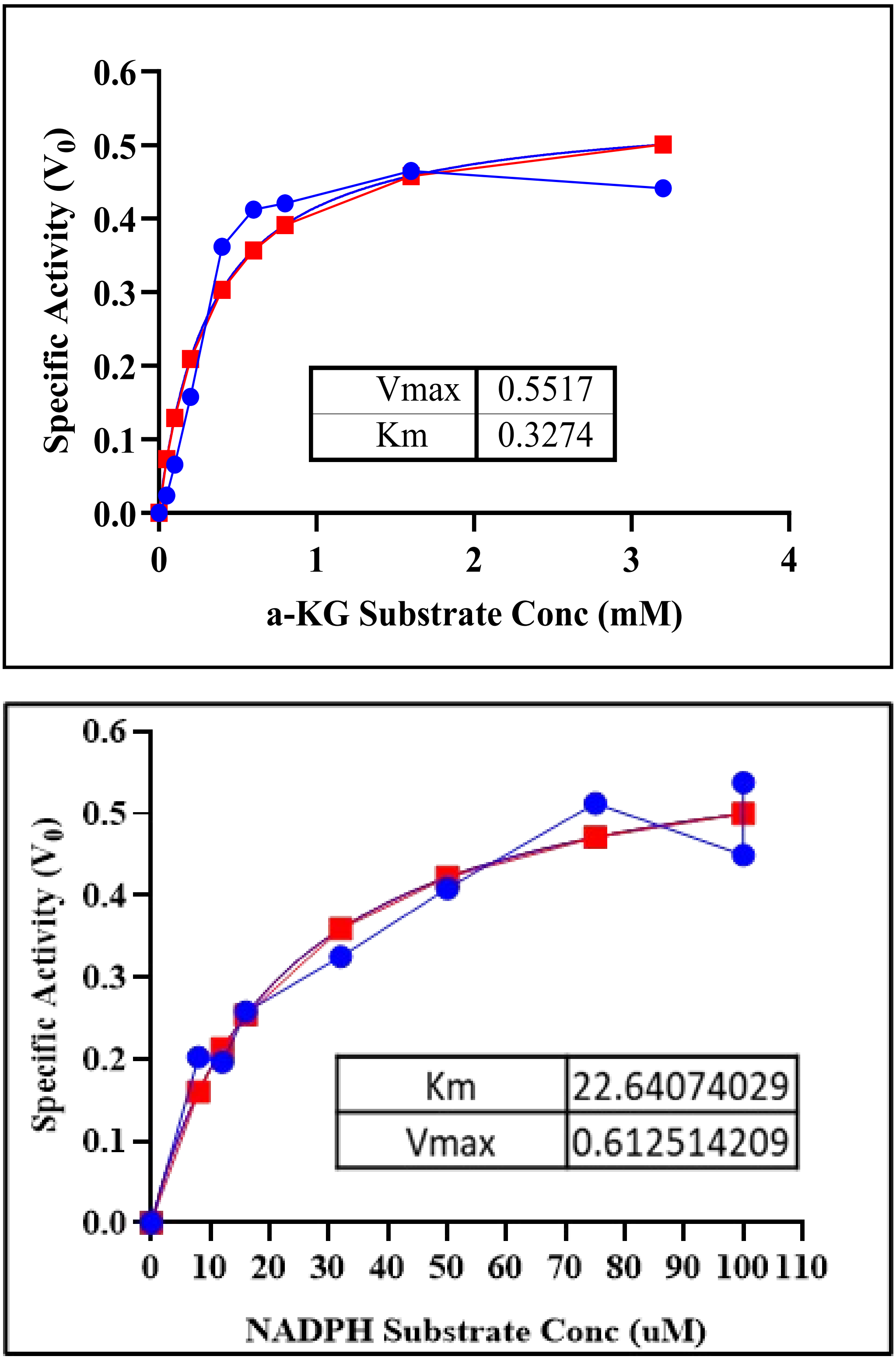

Supplement: RA-015-D5RA06290J-s004 [file RA-015-D5RA06290J-s004.pdf]
